# Antimicrobial Resistance in Food Animals and the Environment in Nigeria: A Review

**DOI:** 10.3390/ijerph15061284

**Published:** 2018-06-17

**Authors:** Nurudeen Olalekan Oloso, Shamsudeen Fagbo, Musa Garbati, Steve O. Olonitola, Emmanuel Jolaoluwa Awosanya, Mabel Kamweli Aworh, Helen Adamu, Ismail Ayoade Odetokun, Folorunso Oludayo Fasina

**Affiliations:** 1 Department of Production Animal Studies (Epidemiology section), Faculty of Veterinary Science, Onderstepoort Campus 0110, University of Pretoria, 0110, South Africa; 2 Public Health Agency, Ministry of Health, Riyadh, 11176, Saudi Arabia; oloungbo@yahoo.com; 3 Department of Medicine, Infectious Diseases and Immunology Unit, University of Maiduguri, PMB 1069, Maiduguri 600230, Borno State, Nigeria; musagarbati@gmail.com; 4 Department of Microbiology, Faculty of Life Sciences, Ahmadu Bello University, Zaria 810241, Nigeria; olonisteve@yahoo.com; 5 Department of Veterinary Public Health and Preventive Medicine, University of Ibadan, Ibadan 200284, Nigeria; emmafisayo@yahoo.com; 6 Veterinary Drugs/Animal Welfare Branch, Quality Assurance and Standards Division, Department of Veterinary & Pests Control Services, Federal Min. of Agric. & Rural Dev. F.C.D.A, Area 11, Garki, Abuja 900001, Nigeria; mabelaworh@yahoo.com; 7 Center for Clinical Care and Clinical Research, Plot 784, By Glimor Engineering, Off Life camp, Gwarimpa Express Way, Jabi, Abuja 240102, Nigeria; hadamu@cccr-nigeria.org; 8 Department of Veterinary Public Health and Preventive Medicine, Faculty of Veterinary Medicine, University of Ilorin, Ilorin 240272, Kwara State, Nigeria; odetokun.ia@unilorin.edu.ng; 9 Emergency Centre for Transboundary Diseases (ECTAD-FAO), Food and Agricultural Organization of the United Nation, Dar es Salaam 0701072, Tanzania

**Keywords:** antimicrobial resistance, antibiotics residue, food animals, environment, bacteria, Nigeria

## Abstract

Antimicrobial resistance (AMR) has emerged as a global health threat, which has elicited a high-level political declaration at the United Nations General Assembly, 2016. In response, member countries agreed to pay greater attention to the surveillance and implementation of antimicrobial stewardship. The Nigeria Centre for Disease Control called for a review of AMR in Nigeria using a “One Health approach”. As anecdotal evidence suggests that food animal health and production rely heavily on antimicrobials, it becomes imperative to understand AMR trends in food animals and the environment. We reviewed previous studies to curate data and evaluate the contributions of food animals and the environment (2000–2016) to the AMR burden in Nigeria using a Preferred Reporting Items for Systematic Reviews and Meta-Analyses (PRISMA) flowchart focused on three areas: Antimicrobial resistance, residues, and antiseptics studies. Only one of the 48 antimicrobial studies did not report multidrug resistance. At least 18 bacterial spp. were found to be resistant to various locally available antimicrobials. All 16 residue studies reported high levels of drug residues either in the form of prevalence or concentration above the recommended international limit. Fourteen different “resistotypes” were found in some commonly used antiseptics. High levels of residues and AMR were found in food animals destined for the human food chain. High levels of residues and antimicrobials discharged into environments sustain the AMR pool. These had evolved into potential public health challenges that need attention. These findings constitute public health threats for Nigeria’s teeming population and require attention.

## 1. Introduction

The reliance of public health and animal health on antimicrobials since the last century is well known and undisputable [1]. Paradoxically, this reliance (sometimes, over-reliance) and its attendant successes have evolved to become a threat to global animal and human health through the phenomenon of antimicrobial resistance (AMR) [2]. Following the development and use of an antimicrobial, various pathogens, in their attempt to survive or evade current and new antimicrobials, undergo evolutionary processes, which results in a short to long term resistance [3]. AMR is the ability of a microorganism (bacteria, viruses, and certain parasites) to prevent an antimicrobial (antibiotics, antivirals, and antimalarials) from working against it [4]. This may lead to resultant ineffectiveness of standard treatments and the infections may persist, with a higher likelihood of spread [5]. The World Health Organization (WHO) presented the level of exposure of the challenges of AMR through the report of the general worldwide situation analysis [4]. This magnitude of threat associated with AMR then received the highest level of political commitment from world leaders and was discussed at the United Nations General Assembly in 2016, where a political declaration on AMR was issued [6]. Hitherto, WHO and the Food and Agriculture Organization of the United Nations (FAO) produced some fundamental documents toward curbing the threat of AMR. These include the WHO Global action plan on antimicrobial resistance and the FAO action plan on antimicrobial resistance 2016–2020, respectively [7,8]. The report from the monitoring of the global action plan by FAO has suggested and recommended the need for situation analysis and production of action plans for individual countries [9].

Food producing animals are linked to humans via the food chain and shared environment [10]. Thus, a One Health approach is necessary to study and understand how to control burdens of AMR, including those presented through foodborne transmission routes [11,12], as well as create a sound and broad-based antimicrobial stewardship program worldwide [12].

Nigeria is also confronted with the burdens of AMR. The Nigerian Centre for Disease Control (NCDC), in collaboration with other institutions, has made efforts to develop an approach to combat AMR using an evidence-based method. Meanwhile, NCDC (2017) reported that Nigeria has experienced huge resistance to antimicrobials in humans, especially in sepsis, respiratory, and diarrheal infections. These include childhood-related life-threatening diseases and are supported by empirical evidence, which are replete and scattered in peer-reviewed and grey literature, as well as commissioned reports [13]. In addition, the situation analysis and recommendations on AMR and drug use in Nigeria has recently been documented [13]. This document still requires detailed information about several sources of AMR, creating a gap in the trend, status, and situation of AMR arising from food animals and the environment. This study fills that gap through a systematic review of published studies and available reports. Specifically, the study collates, curates, and analyzes data on AMR in Nigeria related to food producing animals and the environment, and the immediate human link as contributors to the burdens of AMR in Nigeria. This study is required as a reference source towards the development of a good antimicrobial stewardship program by stakeholders through the “One Health Platform” for Nigeria.

## 2. Materials and Methods

### 2.1. Research Question(s)

We developed some research questions that were used as guides during the study to pursue the attainment of our objectives towards establishing the situation analysis of AMR in the Nigerian environment from food animals. What was the status of antimicrobial resistance in the food producing animals and the environment in Nigeria in the previous studies? What was the pattern of resistance among the classes of antimicrobials tested? What was the status of resistance among the common Nigerian antiseptics and disinfectants that sought to control pathogens at the environmental interface? What were the common organisms and their AMR resistance patterns studied in Nigeria to date?

### 2.2. Search Design

We searched specific databases (Pub Med-NCBI, Google Scholar, Cabdirect, Medline, Embase, Cochrane, and African Journals Online) and various institutional repository of Nigeria using broad terms, “antimicrobial, resistance, and Nigeria”. Where necessary, search terms were stated as strings: Antimicrobial resistance OR Antibiotic resistance OR Antibiotic residue OR Antimicrobial susceptibility OR Antibiotic abuse OR Antibiotic misuse AND Nigeria AND animals; “animals” was substituted with environment and different animal names (poultry, goat, sheep, cattle, camel, pig, etc.). References in the identified materials were also searched and contacted. This effort yielded a broad list of 2393 studies from all sources by the contributors. After removing duplicates, we obtained 435 studies, which were screened to 235 studies by excluding studies conducted prior to the year, 2000, and those with Nigerian authors or affiliations, but focused on samples from outside Nigeria. Upon assessment, we obtained 139 publications and a further 80 were excluded to give 59 publications included in the review and analysis. Each publication was treated as a study, which contains single or multiple reports. The 80 studies excluded did not directly relate to the objectives or yielded information that could be subjected to organized peer review and data analysis. The 59 included studies were sorted into three categories of 42 antimicrobial resistance studies [14,15,16,17,18,19,20,21,22,23,24,25,26,27,28,29,30,31,32,33,34,35,36,37,38,39,40,41,42,43,44,45,46,47,48,49,50,51,52,53,54,55], 16 antimicrobial residue studies [56,57,58,59,60,61,62,63,64,65,66,67,68,69,70,71], and 1 antiseptic or disinfectants study [72]. The PRISMA-style flowchart was modified and used for this analytical review (Figure S1) [73].

### 2.3. Analysis

The number of publications (Table 1a), diversity of methods of data reporting, multiple appearances of study populations reported (Table 1b) in each study, and the objectives of the various studies of the 59 publications we reviewed made it expedient to find a system of accommodating the information through a uniform standard for data harmonization and interpretation in line with the objectives of this study. The various methods of data analysis in all the studies were reviewed to form a unified scale as presented in Table 2. This scale was developed to harmonize the diverse data for analyzing the situation of AMR in Nigeria within the 42 antimicrobial resistance studies (AMRS) and 16 antimicrobial residue studies (ARS). Therefore, the data of reported resistance and residue in the studies were categorized and interpreted according to the standard developed (Table 2). Percentage in Table 2 referred to the percentage (portion) of resistant microbe populations (species) per study. The methods used in most studies were descriptive statistics simple percentages. Some ARS reports were presented in relation to the FAO or WHO standard of maximum residue limit (MRL) at the time of publication. In such studies, the report where no residue was found is categorized as “No residue”, the report where there was residue below standards is categorized as “Low residue”, and the report where the mean residue level was above the MRL is categorized as “Very high residue”. Analysis of the data was then done with MS Excel using simple descriptive statistical analysis, pivot tables, and charts.

## 3. Results

We observed that few studies were undertaken before 2009, with no AMRS, but only four ARS, after which there was an increase in AMRS research from 2009 until recently (Table 1a). The study population involved were environment, cattle, poultry, pig, goat, vegetables, human, bats, camel, sheep, and fish listed in descending order of the number of reports and the type of resistance reported (Table 1b). The study populations appeared singly or in multiple in a study (Table 1b). Also, each study reported from one zone or several geopolitical zones of Nigeria (Figures S2 and S3). Our review revealed that these studies on samples from animals and the environment carried out between 2000 and 2016 fell into three categories (Table 1b).

### 3.1. Antimicrobial Resistance Studies (AMRS)

**Figure 1:**
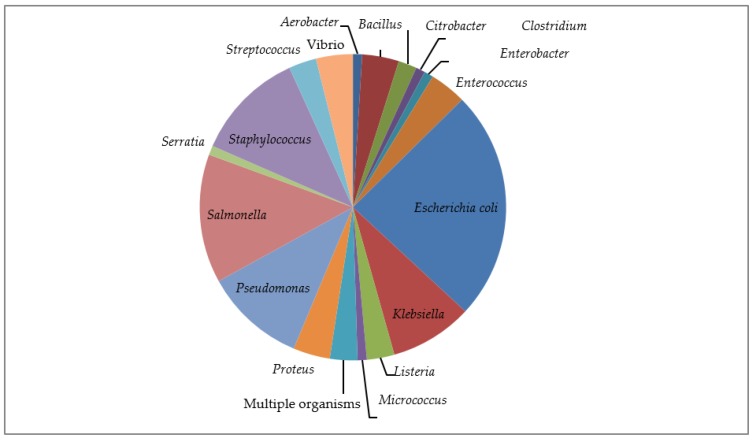
Distribution of organisms studied in the antimicrobial resistance studies based on reports.

**Figure 2:**
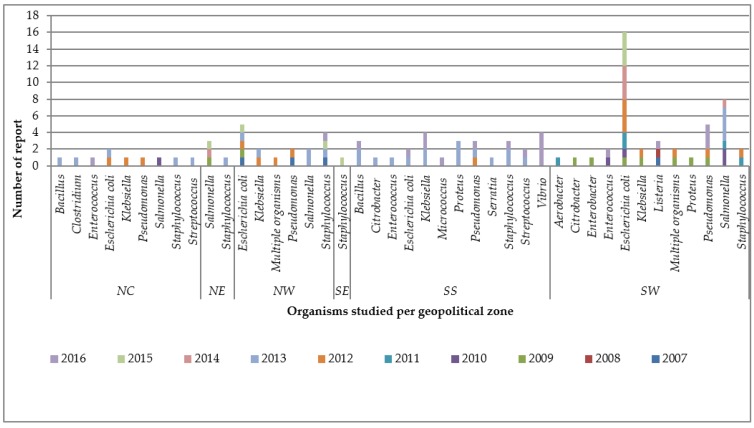
Number of reports yearly per organism for the geopolitical zones of Nigeria. NC = North central, NE = North east, NW = North West, SE = South East, SS = South South, SW = South West.

This category included 42 studies, with the inclusive eligibility criteria in which diverse phenotypic or genotypic methods were utilized ([14,15,16,17,18,19,20,21,22,23,24,25,26,27,28,29,30,31,32,33,34,35,36,37,38,39,40,41,42,43,44,45,46,47,48,49,50,51,52,53,54,55], Tables S1–S3). These studies sought to detect the presence and extent of AMR in collected samples with a selected panel of antibiotics. Cumulatively, these 42 studies tested 68 antimicrobials (Table 3) belonging to different classes and generations of antibiotics from the first to fourth generation of antibiotics, including others that cannot be classified based on generations that were placed on “no generational classification” (NGC) in the course of the analysis (Table 3, Figure S4a,b, and Figure S5a). These resulted in the report of 1139 antimicrobial resistance findings. Out of the 42 studies, only one study on camel samples [45] did not report multidrug resistance (MDR). Two studies [30,38] reported low MDR in cattle and camel samples, and the remaining 39 studies confirmed various patterns of MDR. The AMRS were based on 18 organisms (genus) with species or serovars appearing at least once (Figure 1). The five most important pathogens in which AMR testing was carried out were *E. coli*, *Salmonella* serovars, *Staphylococcus aureus*, *Pseudomonas* spp., and *Klebsiella* spp. *Enterococcus* spp., *Vibrio* spp., *Proteus* spp., and *Listeria* spp. are other microbes used by researchers in AMRS (Figure 1).The nationwide geographical distribution pattern based on geopolitical zones demonstrated that the highest number of reports were from South West Nigeria (44 studies) and, in descending order, from South South (28), North West (16), North Central (10), North East (4), and the lowest was South East (1), which showed poor distribution of studies at the North East and South East (Figure 2, Figure S2).

#### 3.1.1. Antimicrobial Resistance According to Generation of Antibiotics

Antimicrobial resistance within the generational classification of antibiotics used in AMRS (Table 3) revealed that the 68 antibiotics used in all 42 studies involved first, second, third, and fourth generations, and NGC. The generational classification (Table 3) was done using the WHO and the World Organization for Animal Health (OIE) lists of critically important antimicrobial in humans and animals [74,75,76]. This classification is, essentially, based on the spectrum of activity, which increased from first to fourth generation, implying narrow to broad coverage of antibiotics’ action [74]. Cumulatively, of the 1139 antimicrobial report findings, the NGC had the highest number of reports of 537 in the studies of different resistance levels, followed by second and third generation at 210 and 205 reports, respectively; then, fourth generation at 100 and first generation at 86 reports of the resistance findings (Table 3, Figure S4a). The pattern of resistance (Figure S4b) based on proportional percentages of reports showed about 30% of reports on third and fourth generation, and NGC antimicrobials; 20% of first and second generation had very high levels of resistance. It was only 30% of the reports on first, second, and fourth generation, then 20% of third and NGC antimicrobials that had no resistance (Table 3, Figure S4b).

#### 3.1.2. Resistance Level within the Classes of Antibiotics

The 1139 antimicrobial report findings from the 68 antimicrobials included in the panels of all the studies (AMRS) belonged to 19 classes of antibiotics: Aminoglycoside, Ansamycin, Carbolic acid, Diaminopyrimidine inhibitor (DPI), Furan, Glycopeptide, Macrolide, Organophosphate, Oxazolidinone, Phenicol, Polypeptide, Quinolone, Steroid, Streptogramins, Sulfonamides, Sulfonamides + Diaminopyrimidine combinations (SDPI), Tetracycline, β-lactam, and β-lactam + β-lactamase inhibitor combination (Table 3 and Table 4). The number of appearances along the resistance level of these classes (Table 4) revealed β-lactam, Quinolone, and Aminoglycoside as the predominant classes studied. The distribution of these classes along the generation showed that β-lactam derivatives, Quinolone, polypeptide, and streptogramins were the antibiotics with generational classification, while others fall in NGC (Table 3 and Table 4, Figure S5a,b). Therefore, the distribution of resistance within them have great connected implications in human health as they are mostly used in treating disease conditions in hospitals [76].

Using the developed standard (Table 2), we observed the distribution pattern of resistance levels within classes (Table 4) demonstrated that polypeptides and carbolic acids were the only classes where organisms studied had all the reports to be the “no resistance” category (Table 4). Oxazolidinone, Ansamycim, streptogramins, and Aminoglycosides antibiotics were, at best, categorized as “very low resistance”. Meanwhile, phenicol, β-lactam DPI, SDPI, furan, glycopeptides, macrolides, organophosphate, and tetracycline were, at best, of the “very high resistance” category. The highest level of resistance within the resistance pattern distributions among the antibiotic classes were in steroids and sulfonamides, with 70% of the reports on them having “high resistance” to “very high resistance” (Table 4, Figure S5a,b). Each class had peculiar patterns of resistance among the antibiotics belonging to them, which is important for further exposure of the situation of AMR.

##### β-lactam Derivatives

**Figure 3:**
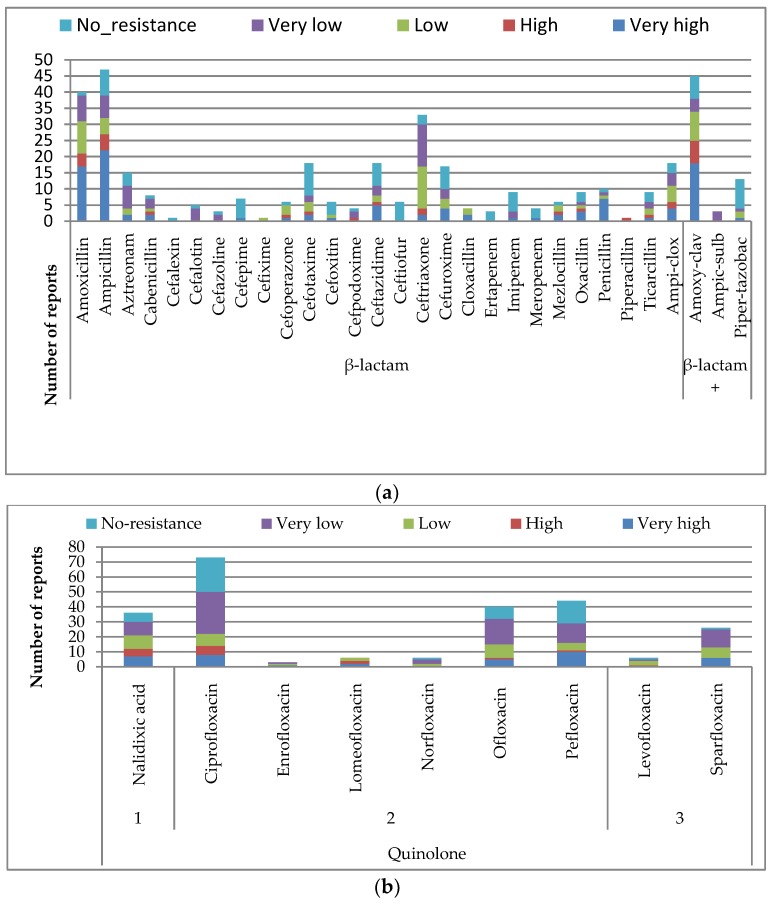

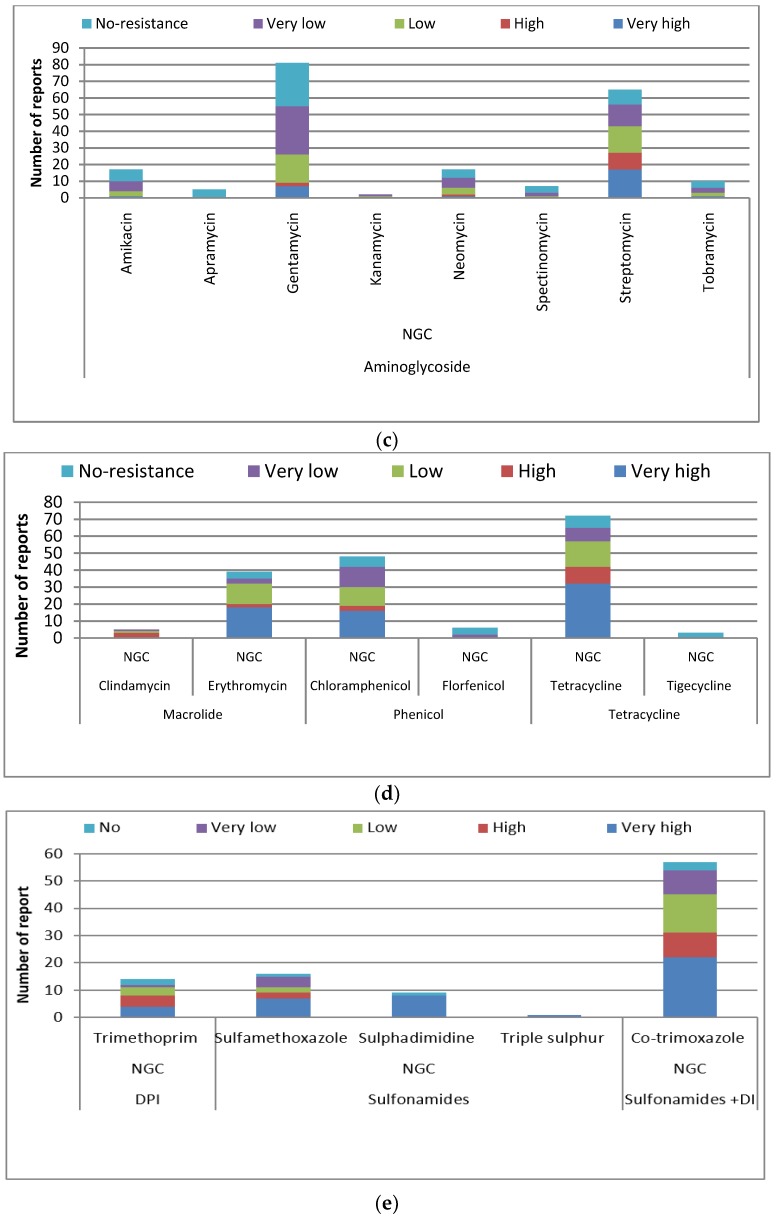
Number of reports of antimicrobial resistance levels of (**a**) β-lactam derivatives; (**b**) Quinolones, (**c**) Aminiglycosides; (**d**) Macrolides, Phenicols, and Tetracyclines; and (**e**) Sulfonamides derivatives antimicrobials.

These were the most tested, constituting 32.4% of all classes of antimicrobials in this study (Table 4). The β-lactam combinations consisted of β-lactam 27% and β-lactam combinations (β-lactam and β-lactamase inhibitors) at 5.4%. The combinations were supposed to improve the sensitivity of the antibiotics against resistant organisms. However, in this study, the organism tested demonstrated higher levels of resistance to β-lactam combinations (19/61) over β-lactam (80/308), which reported very high resistance levels (Table 4, Figure 3a, Figure S6). We observed Amoxycillin-clavunalic acid as one of the most studied β-lactam derivatives, with organisms showing the highest resistance levels to it among the β-lactam combinations, while Piperacillin-tazobactam was the most sensitive, with a lesser proportion of reports of resistance among β-lactam combinations (Table 3 and Table 4, Figure 3a). Among the β-lactams, the third generation antibiotics were the most researched, with Ampicillin and Amoxycillin highest in study rate and also with the highest number of reported resistance, with above 50% of reports on them having very high resistance (Figure 3a, Figure S6). Among all β-lactam derivatives, cefalexin in second generation, Ceftioufur in third generation, and ertapenem in NGC were the only antimicrobials that had all reports on them to be “no resistance” (Figure 3a, Figure S6). All other β-lactams had various patterns of resistance level.

##### Quinolones

This was the second most studied (21.1%) class of antibiotics (Table 4). It comprised nine antimicrobials, with Ciprofloxacin as the most studied. Lomeofloxacin, of the second generation antibiotics, had the highest resistance level, with over 65% of its reports being “high resistance” to “very high resistance” (Figure 3b, Figure S7). The pattern of resistance had little difference along the generation within this class.

##### Aminoglycosides

These constituted 17.77% of the studied antibiotics (Table 4), with gentamycin and streptomycin dominating the antibiotics researched in this group. Streptomycin had the highest level of resistance from organisms tested, with a proportion of 40% of its report to be “high to very high resistance” (Figure 3c, Table 3 and Table 4, Figure S8). Apramycin was the only antibiotic that was not resisted; all reports on it had “no resistance”, while spectinomycin had 80% of its reports with no resistance. The antibiotics in this class demonstrated various patterns of resistance levels (Figure 3c, Figure S8).

##### Macrolide, Phenicol, and Tetracycline

All these three classes belonged to the NGC. Tetracycline, chloramphenicol, and erythromycin dominated, in descending order, respectively. Tetracycline had the highest level of resistance, with 58% of its report to be “high” to “very high resistance” from the organisms researched. It was followed by erythromycin (50%) and chloramphenicol (40%) had “high” to “very high resistance”, then clindamycin, with 60% of reports on it being “high resistance”. Tigercycline was the only one that had all the reports on it as “no resistance” and florfenicol, with 65% as no resistance (Figure 3d, Figure S9).

##### Sulfonamides Derivatives

All the sulfonamides studied belonged to NGC. The three classes and antibiotics studied were Diaminopyrimidine inhibitor (Trimethroprim), Sulfonamides (sulfamethoxazole, sulphadimidine, and triple sulphur), and sulfonamides-diaminopyrimidine inhibitor combination (co-trimoxazole). The reported proportional resistance level in these classes of antibiotics was the most heightened. The combination (co-trimoxazole) was the most studied and 55% of the studies on it reported “high” to “very high resistance level” from organisms studied. The triple sulphur had only one report and the study reported “very high resistance” level to it. Sulphadimidine had eight out of nine reports (90%) to be “very high resistance level”, while trimethoprim and sulfamethoxazole both had 55% that reported a “high” to “very high resistance” level (Figure 3e, Table 4, Figure S10).

##### Other Classes of Antibiotics

The other classes contributed a minute number of report findings, with each class consisting of one antibiotic only; hence, they were pooled together for analysis. Among them, nitrofuran was the most studied and had a high resistance level like vancomycin and fosfomycin, with 50% of the reports having a “high” to “very high resistance” from the organisms studied. In this group, colistin and mupirocin were the most sensitive because they had all reports on them as the “no resistance” level; rifampicin had all its report as “very low resistance”, while teicoplan had the highest resistance, with all the reports on it as “very high resistance” from organisms studied. Then fusidic acid responded to the isolates, with about 70% of the reports to be ”very high resistance” (Figures S11 and S12).

#### 3.1.3. Resistance along the Organisms Studied

The AMRS were based on 18 organisms (genus), with species or serovars appearing at least once (Figure 1). The organisms’ appearance, in descending order, were: *Escherichia coli*, *Salmonella*, *Staphylococcus*, *Pseudomonas*, *Klebsiella, Bacillus*, *Enterococcus*, *Proteus*, *Vibrio*, *Listeria*, *streptococcus*, *Citrobacter*, *Aerobacter*, *Clostridium*, *Enterobacter*, *Micrococcus*, and *Serratia* (Figure 1). The distribution of the organisms studied yearly at geopolitical zones demonstrated some organisms were studied more in particular regions or geopolitical zones of Nigeria (Figure 2).

##### *Escherichia coli* (*E. coli*)

**Figure 4:**
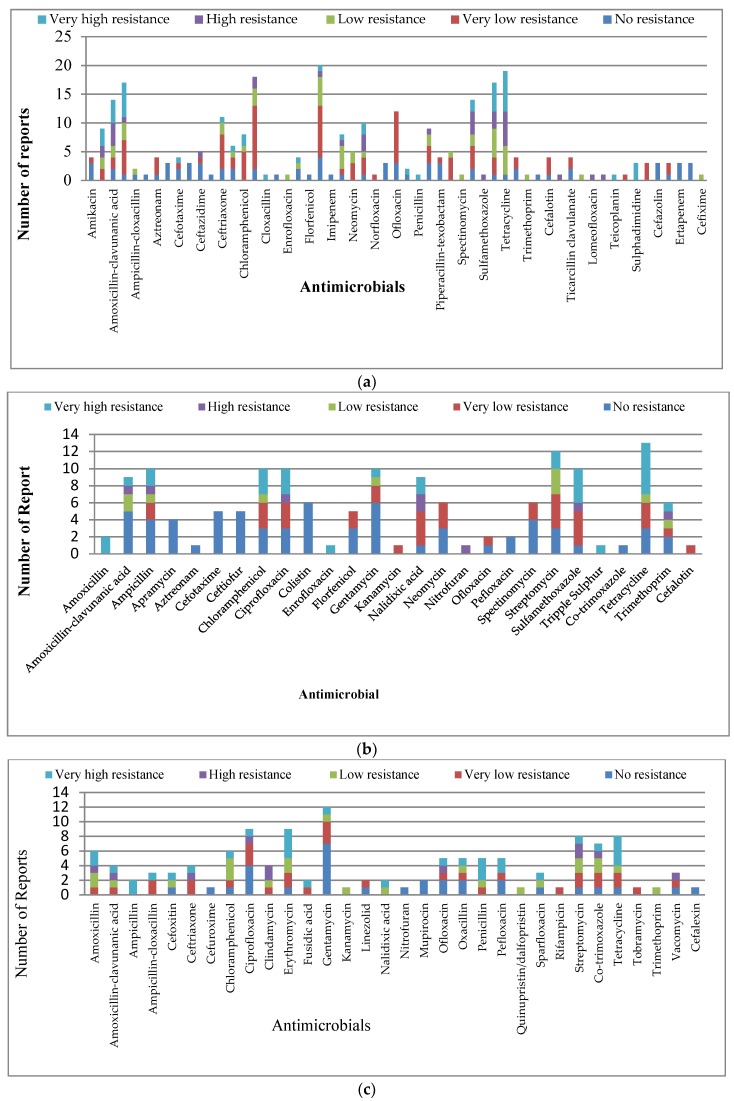

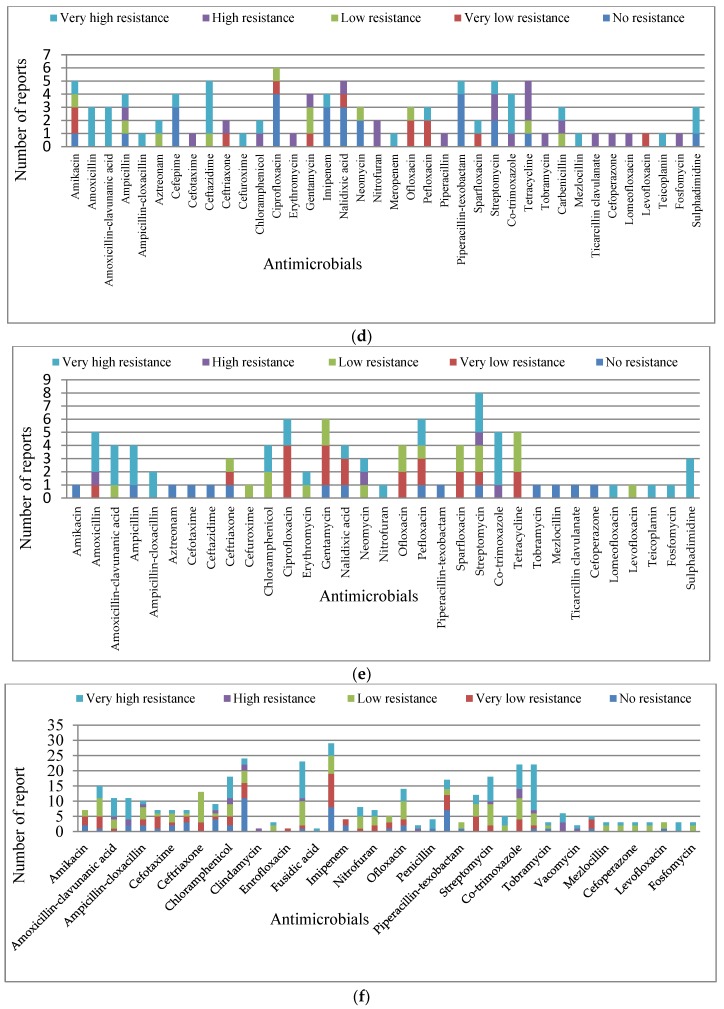
Number of reports of antimicrobial resistance categories for (**a**) *Escherichia coli*; (**b**) *Salmonella*; (**c**) *Staphylococcus*; (**d**) *Pseudomonas*; (**e**) *Klebsiella*; and (**f**) other bacteria.

It was the most studied organism (25%) in Nigeria, but had a skewed distribution, with a higher concentration of *E. coli* studies in South West Nigeria and none in the North East and South East (Figure 2). The distribution of the studies revealed that 57 antibiotics were used to test AMR in *E. coli* isolates, with gentamycin, tetracycline, ciprofloxacin, cotrimoxazole, ampicillin, streptomycin, amoxicillin-clavulanic acid, ofloxacin, ceftriaxone, nitrofuran, perfloxacin, amoxicillin, nalidixic acid, chloramphenicol, cefuroxime, ceftazidime, neomycin, and sparfloxacin being the most prominent in descending order, respectively (Figure 4a). All the reports on *E. coli* isolates revealed “no resistance” to Apramycin, cefepime, cefoxitin, ceftiofur, colistin, florfenicol, Imipenem, meropenem, vacomycin, cefazoline, ertapenem, and tigecycline in the studies that incorporated into the panel of antimicrobial tested. The *E. coli* isolates researched showed “very-low resistance” to “no-resistance” levels in some antibiotics: Amikacin, aztreonam, norfloxacin, ofloxacin, tobramycin, cefalotin, ticarcillin clavulanate, and cefpodoxime in all reports that used them. However, all reports had a “very high resistance” level to cloxacillin, penicillin, teicoplanin, and sulphadimidine where they were included. We observed other various patterns of resistance levels to the remaining antibiotics studied (Figure 4a, Figure S13).

##### *Salmonella* 

It was the second most studied organism (14%) in all geopolitical zones, except the South East and South South where there were none (Figure 1 and Figure 2). The distribution of the studies revealed that 27 antibiotics were used to test for AMR in *Salmonella* isolates, with a close distribution in the number of appearances of individual antibiotics (Figure 4b). The pattern of resistance reported showed that *Salmonella,* studied in all the reports, had no resistance to apramycin, aztreonam, cefotaxime, ceftiofur, colistin, pefloxacin, and co-trimoxazole. We observed that florfenicol, neomycin, ofloxacin, and spectinomycin, respectively, had 40%, 50%, 50%, and 30% of the report on them to be “very low resistance”, but had the remaining 60%, 50%, 50%, and 70% of their reports as the “no resistance” category. Cefalotin and kanamycin had all their reports as the “very low resistance” category. However, all reports on amoxicillin, enrofloxacin, and triple sulphur had “very high resistance”. Other various patterns of resistance were observed in the remaining antibiotics studied (Figure 4b, Figure S14).

##### *Staphylococcus* 

This genus was the third most studied (12%) pathogen for AMR in Nigeria, with the widest spread across all geopolitical zones (Figure 1 and Figure 2). The distribution of the studies of all antibiotics used revealed that 32 antibiotics were used to test the AMR of *Staphylococcus* isolates (Figure 4c). The pattern of resistance reported for *Staphylococcus* showed that all studies that tested cefuroxime, nitrofuran, mupirocin, and cefalexin revealed “no resistance”. All that tested rifampicin and tombromycin reported “very low resistance”. Only two studies reported on linezolid, with one each of “very low resistance” and “no resistance”, and the only study that tested trimethoprim on *Staphylococcus* showed “low resistance” (Figure 4c). However, the two reports on ampicillin had “very high resistance” for *Staphylococcus* isolates. Other patterns (mixed) for the remaining antibiotics tested were observed (Figure 4c, Figure S15).

##### *Pseudomonas* 

This represents the fourth most studied organism (11%) for AMR in Nigeria and had a spread similar to *E. coli* research (Figure 1 and Figure 2). The distribution of the studies of all antimicrobials used revealed that 38 antimicrobials were used to test the AMR in *Pseudomonas* isolates (Figure 4d). Unlike other organisms, there were no antibiotics from the 38 tested with *Pseudomonas* without resistance (Figure 4d). There was “very high resistance” by all *Pseudomonas* studied to amoxicillin, amoxicillin-clavunanic acid, ampicillin-cloxacillin, cefuroxime, meropenem, mezlocillin, and teicoplanin and “high resistance” to cefotaxime, erythromycin, nitrofuran, piperacillin, tobramycin, ticarcillin clavulanate, cefoperazone, lomeofloxacin, and fosfomycin (Figure 4d). All reports of studies that tested chloramphenicol with *Pseudomonas* spp. had 75% of them to be “very high resistance” and the remaining 25% of reports were “high resistance”. Various resistance patterns were observed in the remaining antibiotics studied (Figure 4d, Figure S16).

##### *Klebsiella* 

This is the fifth most studied organism and contributed 9% of the overall studies for AMR in Nigeria, with spread across four out of the six geopolitical zones (South West, South South, North West, and North Central) of Nigeria (Figure 1 and Figure 2). The distribution pattern of the appearance of all antimicrobials used revealed that 33 antimicrobials were used to test the AMR of *Klebsiella* isolates (Figure 4e). All the *Klebsiella* spp. studied demonstrated “no resistance” to amikacin, aztreonam, cefotaxime, ceftazidime, piperacillin-texobactam, tobramycin, mezlocillin, ticarcillin clavulanate, and cefoperazone and “low resistance” to cefuroxime and levofloxacin; but, “very high resistance” to ampicillin-cloxacillin, nitrofuran, lomeofloxacin, teicoplanin, fosfomycin, and sulphadimidine (Figure 4a). Meanwhile, it demonstrated a high proportion of “very high resistance” in amoxicillin (60%), amoxicillin-clavunanic acid (75%), ampicillin (75%), chloramphenicol (50%), erythromycin (50%), neomycin (33%), and co-trimoxazole (80%) (Figure 4e, Figure S17).

##### Other Organisms

All other organisms that made minute contributions were pooled together for analysis. They were spread across the four geopolitical zones of South West, South South, North West, and North Central of Nigeria (Figure 1 and Figure 2). Analysis revealed 43 antimicrobials were used to test for AMR in these organisms (Figure 4f). The organisms were *Proteus, Listeria, Enterococcus, Enterobacter, Citrobacter, Aerobacter, Vibrio, Streptococcus, Serratia, Micrococcus, Bacillus,* and *Clostridium* (Figure 1). All of them had “very high resistance” to fusidic acid and teicoplanin; “high resistance” to clindamycin; but, “very low resistance” to enrofloxacin (Figure 4f). However, they had resistance levels that were “very high resistance” and “high resistance” (combined) to some popular antimicrobials in Nigeria: Amoxicillin (30%), amoxicillin-clavunanic aicd (65%), ampicillin (82%), ampicillin-cloxacillin (20%), aztreonam (15%), cefotaxime (15%), ceftazidime (15%), cefuroxime (35%), chloramphenicol (50%), ciprofloxacin (15%), cloxacillin (30%), erythromycin (55%), nalidixic acid (40%), nitrofuran (30%), ofloxacin (30%), Oxacillin (100(50/50)%), penicillin (75%), perfloxacin (20%), sparfloxacin (25%), streptomycin (50%), sulfamethoxazole (60%), co-trimoxazole (50%), tetracycline (75%), tobramycin (35%), trimethoprim (100(50/50)%), vacomycin (100(50/50)%), carbenicillin (20%), mezlocillin (30%), ticarcillin clavulanate (30%), cefoperazone (30%), lomeofloxacin (30%), and fosfomycin (35%) (Figure 4f, Figure S18).

### 3.2. Antimicrobial Residue Studies (ARS)

**Figure 5:**
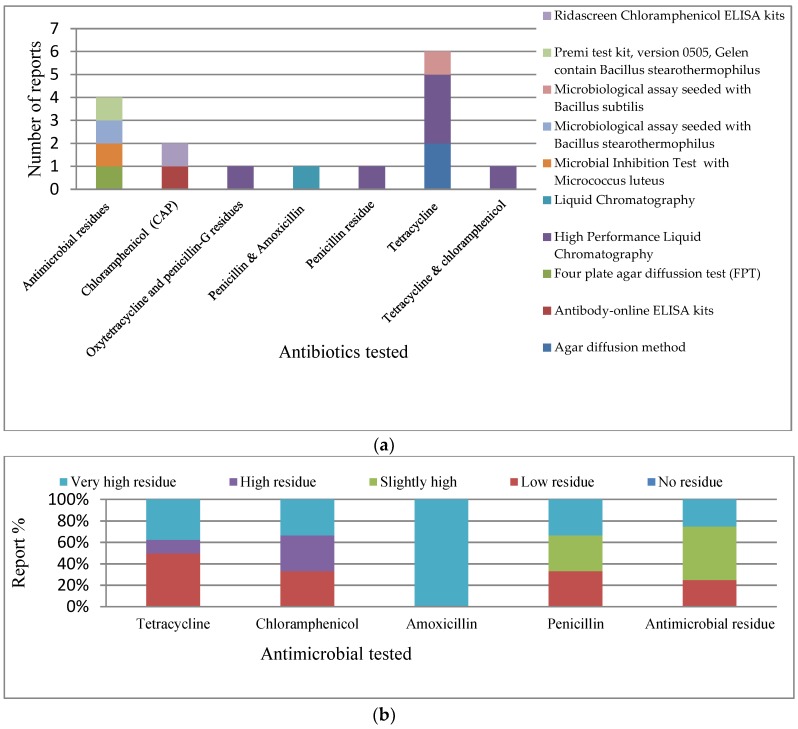
(**a**) Test procedure for each antibiotic tested in antimicrobial residue studies; (**b**) Relative level of antimicrobial residue. Tetracycline: Very high residue (*n* = 3), High (*n* = 1), Low (*n* = 4); Chloramphenicol: Very high (*n* = 1), High (*n* = 1), Low (*n* = 1); Amoxicillin No: (*n* = 1); Penicillin: Very high (*n* = 1), Slight high (*n* = 1), Low (*n* = 1); and Antimicrobial residue generally: Very high (*n* = 1), Slightly high (*n* = 2), Low (*n* = 1).

Summarized in Table 5, in this category, 16 studies were identified that dealt with antimicrobial residues in animals and the environment between 2000 and 2016. We considered published research involving qualitative and quantitative assessment of antimicrobial residues in tested samples. We observed the geographical spread of the studies in this category was poor and was skewed to the South West of Nigeria, with few studies in the South East, North Central, and North West, and no studies from the North East and South South (Table 5, Figure S3). The test procedures utilized by the researchers in the studies included microbiological assay (MA), immunological assay, and chromatography. Specifically, the Ridascreen chloramphenicol ELISA kits, Premi test kit (version 0505, Gelen contain *Bacillus stearothermophilus*), MA (seeded with *Bacillus subtilis*), MA (seeded with *Bacillus stearothermophilus*), microbial inhibition test (contain *Micrococcus luteus*), liquid chromatography, High Performance Liquid Chromatography (HPLC), four plate agar diffusion test (FPT), antibody-online ELISA kits, and the agar diffusion method was used (Table 5, Figure 5a). The reference drugs used for the measurement of residue in all studies singly or in pairs were penicillin, amoxicillin, oxytetracycline, and chloramphenicol, and some researchers only measured antimicrobial residue without mentioning a specific drug (Table 5, Figure 5a). Using a unified scale developed (Table 2), no study revealed “No residue”; while they all reported different levels of residue (Table 5, Figure 5a,b). Tetracycline demonstrated to be the most researched (Figure 5a), with reports demonstrating about 40% as a “Very high residue” level (Figure 5b). Other antibiotics demonstrated lower “Very high residue” levels, with the exception of amoxicillin as shown in Figure 5a,b (Table 5, Figure 5b).

### 3.3. Antiseptics and Disinfectant Chemicals

Only one study identified human and chicken isolates of *Campylobacter jejuni* to show resistance to at least 19 different commonly used chemicals to control microbes [72].

## 4. Discussion

We found that several patterns of multidrug resistance were reported in the different studies reviewed and confirmed high levels of resistance to various antimicrobials and common chemical agents [76,77,78,79]. mostly used in Nigeria for prophylactic and therapeutic purposes in animals, as well as for the control and management of multiple bacterial pathogens encountered in veterinary and human medical environments [76]. These corroborated the reports of some researchers that antibiotics were readily available over the counter (without prescription) against the existing legislation, prompting a very high level of self-medication [77].

The geographical spread of the reviewed studies showed that the problem of AMR is developing nationwide despite increased awareness demonstrated by the number of studies over time. Few human samples were involved in this study where the researchers collected samples from humans along with other samples without separating the result based on sample population. The results in this study, therefore, reflected an interaction with humans. The overall outcome is an indication towards the situation in humans. However, a similar evaluation in the human health system like the current study had revealed that *Escherichia coli*, *Shigella*, *Salmonella Typhimurium*, and *S. Enteritidis* were more isolated in human diagnostic samples, with evidence of zoonotic infections [78]. Patterns of antimicrobial resistance in humans are similar to what we have also established in animal populations and the environment as indicated in this work. Resistance to penicillin, tetracycline, ampicillin, nalixidic acid, chloramphenicol, and cotrimaxole, among others, has been established in humans [78]. Whether the patterns in humans, animals, and the environment have some association cannot be established in this study, but anecdotal evidence suggests that food animals are often slaughtered and pass into the human food chain without the establishment of residual antimicrobials. We found from observation of study populations that camels were relatively free compared to other animals, but this is only in one study. We are careful to make deductions in this regards as a single study may be tricky to make predictions on the level of antimicrobials in camels, although field situation does not support the widespread use of antibiotics in camels. Also, other studies also demonstrated very low levels of AMR in camels. These studies suggested that the situation of AMR reported may be from the predictor of production management because the herders rarely use antimicrobials in camels in comparison to their use in other food animals [38,44].

*Staphylococcus* was the only organism included in all studies in all geopolitical zones and had the widest spatial spread. Therefore, the analysis of studies on *Staphylococcus* had the greatest national reflection of the situation of AMR in Nigerian food animals and the environment. All studies on *Staphylococcus* reported very high levels of resistance to ampicillin. This corroborated the report that ampicillin and its combinations were the most consumed over-the-counter (self-medicated) drugs by humans and in animals in Nigeria [77]. This is of great concern because ampicillin is a third generation and ampicillin-cloxacillin is a fourth generation, both of β-lactams. Although, *E. coli* was the most studied, *Pseudomonas* spp. had the highest AMR because this pathogen demonstrated resistance to all antibiotics tested. Also, *Salmonella* demonstrated greater AMR than *E. coli.*

Observations of AMR within the classes of antibiotics along the generation reflected higher percentages of resistance in the antibiotics belonging to the β-lactam derivatives and quinolones of the third and fourth generation, and aminoglycosides. This raised further concerns of the threat posed by AMR. These concerns are heightened as these drugs are listed by WHO as critically important antibiotics required in the management of severe disease conditions. Considering the concerns raised by the drop in global inventions and lack of advances in the production of new antibiotics in the last three decades, which has necessitated monitoring of the circulation of antibiotics worldwide, this current situation is critical. WHO, in response to the above, produced and categorized all antibiotics, which is regularly updated yearly. Therefore, the heightened concerns are necessary to stimulate the Nigerian government and the “One Health Platform”, which is under formation, to be proactive towards monitoring, improving, and controlling the current trend. 

The reported rate of “high to very high level” of residue levels in the ARS is a confirmation of the demonstration of resistance levels in the AMR studies. All reference drugs tested in the ARS are commonly used in human and food animals in Nigeria [79]. Very high levels of drug residue in goats’ milk (100%) is of concern. This portends a problem of AMR of food origins in humans [62]. Meanwhile, the high drug residues in Nigeria food delicacies, including muscle, liver, kidney, and milk, means that human exposure risk is high.

High level of resistance implies that most antibiotics are insensitive to most pathogens in the Nigerian environment. This has also affected antimicrobials’ use as antiseptics. These high levels of residues and AMR found in food animals consumed by humans and discharged into environments sustain the AMR pool in addition to the observed resistance by chemicals commonly used as antiseptics to control infection at the point of entrance. This portends a high potential risk to public health management and necessitates the establishment of an institutionalized system that will establish, monitor, control, and promote good antimicrobial stewardship using a one health approach to reduce the current spread of antimicrobial resistance.

Finally, high levels of multiple antibiotic resistance have been observed against many microbial organisms affecting humans and animals. However, most of the studies conducted to date do not use international standards in the delivery of the research results. Future research, in this regard, must carefully consider global standards as part of their methods to engage in carrying out research in Nigeria.

## 5. Limitations

It was difficult to harmonize our results based on the various AMRS approaches used in studies available for assessment. Moreover, in many of the studies, the Kirby-Baeur method was used, but adequate reference to standards from either Clinical & Laboratory Standards Institute (CLSI) or the European Committee on Antimicrobial Susceptibility Testing (EUCAST) was not provided.

Studies on antibiotics residues were scarce in Nigeria during the years under consideration, with limited studies available for analysis. The methods in most of the studies considered commercial kits, with a dearth of in-depth information on the procedures, which should have given ample opportunity to compares the biases in the methods used in the various residue studies. Relevant equipment that should support such studies on residue testing was wanting at the time of these studies in Nigeria.

## 6. Conclusions

Multidrug resistance has shown a heightened rise in Nigeria based on this study. The need to use international standards to evaluate most studies on AMR nationwide, in view of the variance of these standards, is necessary. Most of the antimicrobials observed in this study are on the WHO 2017 list of essential antimicrobials and are also listed in OIE 2017 Terrestrial animal health code has, thus, necessitated the evaluation of the situation of AMR in humans [76,80].

It is necessary to design a carefully planned, multi-sectoral, surveillance plan, which can be adopted for research and diagnostic purposes in various aspect of AMR. The need for standardization in all studies in the future and, possibly, the development of guidelines that should harmonize studies across platforms using the “One Health Approach” is imperative. This should target the promotion of good practices and antimicrobial stewardship, which should be enforced by the government, with the cooperation of all stakeholders

The relevant ministries and government departments should enforce: Registration and monitoring of animal production premises, especially, food producing animals; improvement of biosecurity compliance of food animal environments; prohibition of the use of antibiotics for growth promotion and prophylactic treatment; and putting in place a system to implement drug withdrawal periods in food animals.

More detailed descriptions of the results (figures) are available in the Supplementary materials, which are available online.

## Figures and Tables

**Table 1:** Rate of publication per year (**a**) and population groups identified in the studies (**b**).

**a. Rate of Publication per Year**				
**Publication Year**	**AMRS**	**ARS**	**SDA**	**Total Reports**
2001		1		1
2002		2		2
2003			1	1
2005		1		1
2007	2			2
2008	1			1
2009	4			4
2010	4	1		5
2011	2	1		3
2012	6	7		13
2013	7	1		8
2014	4	1		5
2015	5			5
2016	7	1		8
Total	42	16	1	59
**b. Population Groups Identified in the Studies**				
**Sample Population**	**AMRS**	**ARS**	**SDA**	**Total Reports**
Environment	45	-	1	46
Cattle	28	6	-	34
Poultry	26	6	-	32
Pig	10	2	-	12
Goat	6	3	-	9
Vegetables	3	-	-	3
Human	3	-	-	3
Bats	2	-	-	2
Camel	2	-	-	2
Sheep	2	-	-	2
Fish	1	1	-	1
Total	128	18	1	146

AMRS: Antimicrobial resistance studies. ARS: Antimicrobial residue studies. SDA: Surface disinfectants and antiseptics. Table 1: This is a table to show the number of studies for different measurement parameters: (**a**) Showed the number of studies on each measured parameter for each year; and (**b**) showed the total number of reports of appearance of each population group for each measurement parameter.

**Table 2:** Categorization for the measure of resistance or residue level.

Group Scale	Categorization	Antimicrobial Resistance Studies	Antimicrobial Residue Studies
**1**	≤1%	Sensitive or No resistance	No residue
**2**	>1 ≤ 24%	Moderately sensitive or very low resistance	Low residue
**3**	>24 ≤ 50%	Weakly sensitive or Low resistance	Slightly high residue
**4**	>50 ≤ 74%	Low sensitive or High resistance	High residue
**5**	>74%	Very low (no) sensitive or Very high resistance	Very high residue

Table 2: This is a table showing the scale developed to measure the level of resistance or residue in a harmonized form from different diverse measurements from the several studies. Percentage referred to the proportion of resistant microbe populations (species) per study.

**Table 3:** List of antibiotics used and the number of reports of each antimicrobial resistance.

Antibiotics in Peer-Reviewed Studies (n)	Class	Generation	Number of Reports & Category of Resistance Level					
			Very High	High	Low	Very Low	No	Total
Amikacin (AMK) (5)	Aminoglycoside	NGC	1	0	3	6	7	17
Amoxicillin (AMX) (10)	β-lactam	3	17	4	10	8	1	40
Amoxycillin-clavunanic acid (AMC) (23)	β-lactam +	4	18	7	9	4	7	45
Ampicillin (AMP) (20)	β-lactam	3	22	5	5	7	8	47
Ampicillin-cloxacillin (APX) (3)	β-lactam	4	4	2	5	4	3	18
Ampicillin-sulbactam (AMS) (1)	β-lactam +	4	0	0	0	3	0	3
Apramycin (APR) (5)	Aminoglycoside	NGC	0	0	0	0	5	5
Aztreonam(AZT) (5)	β-lactam	1	2	0	2	7	4	15
Cabenicillin (CBN) (3)	β-lactam	4	2	1	1	3	1	8
Cefalexin (CLX) (1)	β-lactam	2	0	0	0	0	1	1
Cefalotin (CLT) (3)	β-lactam	1	0	0	0	4	1	5
Cefazoline (CFZ) (1)	β-lactam	1	0	0	0	2	1	3
Cefepime (CFP) (3)	β-lactam	4	1	0	0	0	6	7
Cefixime (CFX) (1)	β-lactam	3	0	0	1	0	0	1
Cefoperazone (CPZ) (1)	β-lactam	3	1	1	3	0	1	6
Cefotaxime (CTX) (10)	β-lactam	3	2	1	3	2	10	18
Cefoxitin (CXT) (4)	β-lactam	2	1	0	1	0	4	6
Cefpodoxime (CPM) (2)	β-lactam	3	0	1	0	2	1	4
Ceftazidime (CAZ) (6)	β-lactam	3	5	1	2	3	7	18
Ceftiofur (XNL) (6)	β-lactam	3	0	0	0	0	6	6
Ceftriaxone (CRO) (8)	β-lactam	3	2	2	13	13	3	33
Cefuroxime (CXM) (6)	β-lactam	2	4	0	3	3	7	17
Chloramphenicol (CHL) (21)	Phenicol	NGC	16	3	11	12	6	48
Ciprofloxacin (CIP) (30)	Quinolone	2	8	6	8	28	23	73
Clindamycin (CLI) (5)	Macrolide	NGC	0	3	1	1	0	5
Cloxacillin (CXL) (4)	β-lactam	2	2	0	2	0	0	4
Colistin (COL/CT) (7)	Polypeptide	1	0	0	0	0	7	7
Enrofloxacin (ENR) (3)	Quinolone	2	1	0	1	1	0	3
Ertapenem (ETP) (1)	β-lactam	NGC	0	0	0	0	3	3
Erythromycin (E) (17)	Macrolide	NGC	18	2	12	3	4	39
Florfenicol (FFC) (6)	Phenicol	NGC	0	0	0	2	4	6
Fosfomycin (FFM) (1)	Organophosphate	NGC	2	1	2	1	0	6
Fusidic acid (FUA) (3)	Steroid	NGC	2	0	0	1	0	3
Gentamycin (CN/GEN) (33)	Aminoglycoside	NGC	7	2	17	29	26	81
Imipenem (IMP) (4)	β-lactam	NGC	1	0	0	2	6	9
Kanamycin (K) (2)	Aminoglycoside	NGC	0	0	1	1	0	2
Levofloxacin (LVF) (1)	Quinolone	3	0	1	3	1	1	6
Linezolid (LIZ) (2)	Oxazolidinone	NGC	0	0	0	1	1	2
Lomeofloxacin (LMF) (1)	Quinolone	2	2	2	2	0	0	6
Nalidixic acid (NAL) (16)	Quinolone	1	7	5	9	9	6	36
Neomycin (N) (8)	Aminoglycoside	NGC	1	1	4	6	5	17
Nitrofuran (NIT) (8)	Furan	NGC	5	6	4	5	2	22
Norfloxacin (NOR) (3)	Quinolone	2	0	0	2	3	1	6
Meropenem (MPM) (2)	β-lactam	NGC	1	0	0	0	3	4
Mezlocillin (MZC) (1)	β-lactam	4	2	1	2	0	1	6
Mupirocin (MP) (2)	Carbolic acid	NGC	0	0	0	0	2	2
Ofloxacin (OFX) (11)	Quinolone	2	5	1	9	17	8	40
Oxacillin (OX) (7)	β-lactam	2	3	1	1	1	3	9
Penicillin (P) (6)	β-lactam	1	7	0	1	1	1	10
Pefloxacin (PEF) (9)	Quinolone	2	10	1	5	13	15	44
Piperacillin (PPC) (1)	β-lactam	4	0	1	0	0	0	1
Piperacillin-tazobactam (PTB) (4)	β-lactam + β-LI	4	1	0	2	1	9	13
Quinupristin (QUI) (1)	Streptogramins	2	0	0	1	0	0	1
Sparfloxacin (SPF) (4)	Quinolone	3	6	0	7	12	1	26
Rifampicin (RIF) (1)	Ansamycin	NGC	0	0	0	1	0	1
Spectinomycin (SPE) (6)	Aminoglycoside	NGC	0	0	1	2	4	7
Streptomycin (S) (22)	Aminoglycoside	NGC	17	10	16	13	9	65
Sulphadimidine (SDN) (1)	Sulfonamides	NGC	8	0	0	0	1	9
Sulfamethoxazole (SMX) (10)	Sulfonamides	NGC	7	2	2	4	1	16
Triple sulphur (TS) (1)	Sulfonamides	NGC	1	0	0	0	0	1
Co-trimoxazole (COT) (17)	Sulfonamides + DI	NGC	22	9	14	9	3	57
Teicoplan (TCP) (1)	Glycopeptide	NGC	6	0	0	0	0	6
Tetracycline (T) (30)	Tetracycline	NGC	32	10	15	8	7	72
Ticarcillin (TCC) (2)	β-lactam	4	1	1	2	2	3	9
Tigecycline (TGC) (1)	Tetracycline	NGC	0	0	0	0	3	3
Tobramycin (TMN) (3)	Aminoglycoside	NGC	1	0	2	3	4	10
Trimethoprim (TMP) (10)	DI	NGC	4	4	3	1	2	14
Vancomycin (V) (4)	Glycopeptide	NGC	1	2	0	1	2	6
Total (42)			289	100	223	266	261	1139

NGC: No generation classification. 1,2,3 and 4: First and second generation antibiotics, respectively. β-lactam + β-LI : β-lactam + β-lactamase inhibitor. β-lactam + means β-lactam combined with another antibiotics; DI: Diaminopyrimidine inhibitor. Sulfonamides + DI: Sulfonamides + Diaminopyrimidine inhibitor. (n): Number of peer reviewed studies for each antibiotic are placed in bracket after each antibiotic.

**Table 4:** Number of reports of each resistance level category within the classes of antimicrobial in the Antimicrobial resistance studies.

Class of Antimicrobials	Number of Reports of Each Resistance Level Category					
	Very High	High	Low	Very Low	No	Total n (%)
Aminoglycoside	27	13	44	60	60	204 (17.9%)
Ansamycin	0	0	0	1	0	1 (0.09%)
Carbolic acid	0	0	0	0	2	2 (0.18)
DPI	4	4	3	1	2	14 (1.2)
Furan	5	6	4	5	2	22 (1.9)
Glycopeptide	7	2	0	1	2	12 (1.1%)
Macrolide	18	5	13	4	4	44 (3.9)
Organophosphate	2	1	2	1	0	6 (0.5%)
Oxazolidinone	0	0	0	1	1	2 (0.16)
Phenicol	16	3	11	14	10	54 (4.8%)
Polypeptide	0	0	0	0	7	7 (0.6%)
Quinolone	39	16	46	84	55	240 (21.1%)
Steroid	2	0	0	1	0	3 (0.2%)
Streptogramins	0	0	1	0	0	1 (0.08)
Sulfonamides	16	2	2	4	2	26 (2.3%)
Sulfonamides + DI	22	9	14	9	3	57 (5.0%)
Tetracycline	32	10	15	8	10	75 (6.6%)
β-lactam	80	22	57	64	85	308 (27.0%)
β-lactam + β-LI	19	7	11	8	16	61 (5.4%)
Total	289	100	223	266	261	1139 (100%)

DI = Diaminopyrimidine inhibitor β-LI = β-lactamase inhibitor.

**Table 5:** Summary evaluation of antimicrobial residue studies.

Ref.	Sample				Zone	Test Procedure	Positive Tested Antimicrobial Residue Level				
	Population	Type	Size	Site			TET	CHL	AMX	PEN	AR
**56**	Cattle	Liver, kidney & muscle	180	Ogun Lagos	SW	Agar diffusion method	Low (16.63%)	-	-	-	-
**57**	Cattle	Urine	500	Zaria	NW	Microbial Inhibition Test with *Micrococcus luteus*		-	-	-	Low (7.4%)
**58**	Goat and pig	liver, kidney & muscle	360	Ogun Lagos	SW	Agar diffusion method	Low (15.6%)	-	-	-	-
**59**	Poultry	Imported layer birds meat	100	Ogun, Lagos, Oyo	SW	Microbiological assay seeded with B.S 1	Low (14%)	-	-	-	-
**60**	Cattle	Beef	180	Akure	SW	High Performance Liquid Chromatography	High (54.4%)	-	-	-	-
**61**	Poultry	Eggs	35	Enugu	SE	Microbiological assay seeded with B.S 2	-	-	-	-	Slightly high (30–36%)
**62**	Goat	Milk	166	Ibadan,	SW	Liquid Chromatography	-	-	Very high (100%)	Very high (100%)	-
**63**	Poultry	Chicken egg	125	Ibadan	SW	High Performance Liquid Chromatography	Very high >80%	-	-	-	-
**64**	Goat and pig	Muscle, liver & kidney	240	Nsukka	SE	Four plate agar diffusion test (FPT)	-	-	-	-	Slightly high 25–30%
**65**	Cattle	Kidney, Liver, Muscle, Urine	448	Abuja	NC	Premi test kit, version 0505, Gelen contain B.S 2	-	-	-	-	Very high 89.3%
**66**	Poultry	Eggs, muscles, liver, & kidney	168	Ibadan	SW	Ridascreen CHL ELISA kits	-	High	-	-	-
**67**	Fish	Fresh & frozen fish	60	Ibadan	SW	High Performance Liquid Chromatography	Very high	Very high	-	-	-
**68**	Poultry	Frozen chicken	100	Lagos & Ibadan	SW	High Performance Liquid Chromatography	Very high	-	-	-	-
**69**	Cattle	Organs: kidney, liver, muscles	90	South west	SW	High Performance Liquid Chromatography	Low	-	-	Low	-
**70**	Poultry	Chicken eggs	288	Abuja	NC	Antibody-online ELISA kits	-	Low	-	-	-
**71**	Cattle	Dairy products	598	Oyo state	SW	High Performance Liquid Chromatography	-	-	-	Slightly high	-

TET: Tetracycline, CHL: Chloramphenicol, AMX: Amoxicillin, PEN: Penicillin B.S 1: *Bacillus subtilis.* B.S 2: *Bacillus stearothermophilus* AR: Antimicrobial residue.

## Supplementary Materials

The following are available online at http://www.mdpi.com/1660-4601/15/6/1284/s1, Figure S1: Flow chart of the methodological strategy (PRISMA 2009 Flow Diagram), Figure S2: Nigeria geopolitical zonal spread of the AMRS reports, Figure S3: Geopolitical zonal spread of the Antimicrobial Residue reports, Figure S4a: Level of resistance within generation of antimicrobials tested, Figure S4b: Proportional (%) pattern of resistance levels within generation of antimicrobials tested, Figure S5a: Frequency of Antimicrobial Resistance levels of classes of antibiotics, Figure S5b: Antimicrobial resistance patterns within classes along generation of antibiotics, Figure S6: Antimicrobial resistance patterns of β-lactam derivatives antibiotics, Figure S7: Antimicrobial resistance patterns of Quinolones, Figure S8: Antimicrobial resistance patterns of Aminoglycosides, Figure S9: Antimicrobial resistance patterns of Macrolide, Phenicol, and Tetracycline, Figure S10: Antimicrobial resistance patterns of Sulfonamides derivatives, Figure S11: Frequency of antimicrobial resistance levels of other classes of antibiotics, Figure S12: Antimicrobial resistance patterns of other classes of antibiotics, Figure S13: Pattern of antimicrobial resistance of *Escherichia coli,* Figure S14: Pattern of antimicrobial resistance of *Salmonella,* Figure S15: Pattern of antimicrobial resistance of *Staphylococcus,* Figure S16: Pattern of antimicrobial resistance of *Pseudomonas,* Figure S17: Pattern of antimicrobial resistance of *Klebsiella,* Figure S18: Pattern of antimicrobial resistance of other bacteria, Table excel S1: Raw data AMRS, S2: Comprehensive AMRS data, S3: Categorized AMRS data analytical.

## Abbreviations

AMR	antimicrobial resistance
AMRS	antimicrobial resistance studies
AMX	Amoxicillin
AR	Antimicrobial residue
ARS	antimicrobial residue studies
B.S 2	Bacillus stearothermophilus
BS 1	Bacillus subtilis
CHL (CAP)	Chloramphenicol
ELISA	Enzyme-linked immune sorbent assay
FAO	Food and Agriculture Organization of the United Nations
FPT	Four plate agar diffusion test
HPLC	High Performance Liquid Chromatography
MDR	multidrug resistance
NC	North Central
NCDC	Nigerian Centre for Disease Control
NE	North East
NGC	no generational classification
NW	North West
PEN	Penicillin
SDA	Surface disinfectants and antiseptics
SE	South East
SS	South South
SW	South West
TET	Tetracycline
WHO	World Health Organization

## References

[1] Kingston W. (2000). Antibiotics, invention and innovation. Res. Policy.

[2] O’Neill J. (2015). Antimicrobials in Agriculture and the Environment: Reducing Unnecessary Use and Waste.

[3] O’Neill J. (2014). Antimicrobial Resistance: Tackling a Crisis for the Health and Wealth of Nations.

[4] World Health Organization (2015). Worldwide Country Situation Analysis: Response to Antimicrobial Resistance.

[5] O’Neill J. (2016). Tackling Drug-Resistant Infections Globally: Final Report and Recommendations: Final Report.

[6] United Nations Press Release: High-Level Meeting on Antimicrobial Resistance. Proceedings of the 71st General Assembly of the United Nations.

[7] World Health Organization (2015). Global Action Plan on Antimicrobial Resistance.

[8] Food and Agricultural Organization of the United Nation (2016). The FAO Action Plan on Antimicrobial Resistance (2016–2020). http://www.fao.org/3/a-i5996e.pdf.

[9] Food and Agriculture Organization of the United Nations (2016). Monitoring and Evaluation of the Global Action Plan on Antimicrobial Resistance. http://www.fao.org/3/a-i7711e.pdf.

[10] Marshall B.M., Levy S.B. (2011). Food Animals and Antimicrobials: Impacts on Human Health. Clin. Microbiol. Rev..

[11] Parmley J., Leung Z., Léger D., Finley R., Irwin R., Pintar K., Pollari P., Reid-Smith R., Waltner-Toews D., Karmali M. (2012). A holistic approach toward enteric bacterial pathogens and antimicrobial resistance surveillance. Improving Food Safety through a One Health Approach: Workshop Summary.

[12] Leonard C.T., Ward D., Longson C. (2017). Antimicrobial resistance: A light at the end of the tunnel?. Lancet.

[13] Nigeria Centre for Disease Control (2017). Antimicrobial Use and Resistance in Nigeria. Situation Analysis and Recommendations. Produced by Federal Ministries of Agriculture, Environment and Health. Coordinated by Nigeria Centre for Disease Control. http://www.ncdc.gov.ng/themes/common/docs/protocols/56_1510840387.pdf.

[14] Mzungu I. (2007). Isolation, Antibiotic and Heavy Metal Susceptibility Patterns of Some Pathogens from Domestic Dumpsites and Waste Water. Master’s Thesis.

[15] David O.M., Odeyemi A.T. (2007). Antibiotic resistant pattern of environmental isolates of *Listeria monocytogenes* from Ado-Ekiti, Nigeria. Afr. J. Biotechnol..

[16] Adetunji V.O., Adegoke G.O. (2008). Formation of biofilm by strains of *Listeria monocytogenes* isolated from soft cheese ‘wara’ and its processing environment. Afr. J. Biotechnol..

[17] Adelowo O.O., Fagade O.E. (2009). The tetracycline resistance gene tet39 is present in both Gram-negative and Gram-positive bacteria from a polluted river, Southwestern Nigeria. Lett. Appl. Microbiol..

[18] Raufu I., Hendriksen R.S., Ameh J.A., Aarestrup F.M. (2009). Occurrence and characterization of Salmonella Hiduddify from chickens and poultry meat in Nigeria. Foodborne Pathog. Dis..

[19] Muhammad M., Muhammad L.U., Ambali A.G., Mani A.U., Azard S., Barco L. (2010). Prevalence of Salmonella associated with chick mortality at hatching and their susceptibility to antimicrobial agents. Vet. Microbiol..

[20] Ayeni F.A., Adeniyi B.A., Ogunbanwo S.T., Tabasco R., Paarup T., Peláez C., Requena T. (2009). Inhibition of uropathogens by lactic acid bacteria isolated from dairy foods and cow’s intestine in western Nigeria. Arch. Microbiol..

[21] Garba I., Tijjani M.B., Aliyu M.S., Yakubu S.E., Wada-Kura A., Olonitola O.S. (2009). Prevalence of *Escherichia coli* in some public water sources in Gusau municipal, North-western Nigeria. Bayero J. Pure Appl. Sci..

[22] Fashae K., Ogunsola F., Aarestrup F.M., Hendriksen R.S. (2010). Antimicrobial susceptibility and serovars of Salmonella from chickens and humans in Ibadan, Nigeria. J. Infect. Dev. Ctries..

[23] Odeyemi A.T., Dada A.C., Ogunbanjo O.R., Ojo M.A. (2010). Bacteriological, physicochemical and mineral studies on Awedele spring water and soil samples in Ado Ekiti, Nigeria. Afr. J. Environ. Sci. Technol..

[24] Ojo O.E., Ajuwape A.T., Otesile E.B., Owoade A.A., Oyekunle M.A., Adetosoye A.I. (2010). Potentially zoonotic shiga toxin-producing *Escherichia coli* serogroups in the faeces and meat of food-producing animals in Ibadan, Nigeria. Int. J. Food Microbiol..

[25] Adesiji Y.O., Alli O.T., Adekanle M.A., Jolayemi J.B. (2011). Prevalence of *Arcobacter*, *Escherichia coli*, *Staphylococcus aureus* and *Salmonella* species in Retail Raw Chicken, Pork, Beef and Goat meat in Osogbo, Nigeria. Sierra Leone J. Biomed. Res..

[26] Fortini D., Fashae K., García-Fernández A., Villa L., Carattoli A. (2011). Plasmid-mediated quinolone resistance and β-lactamases in *Escherichia coli* from healthy animals from Nigeria. J. Antimicrob. Chemother..

[27] Amosun E.A., Olatoye I.O., Adetosoye A.I. (2012). Antimicrobial resistance in *Escherichia coli*, *Klebsiella pneumonia* and *Pseudomonas aeruginosa* isolated from the milk of dairy cows in three Nigerian cities. Niger. Vet. J..

[28] (2012). Damian. U.I. Antibiotics Susceptibility Studies of Some Bacterial Isolates from Packaged Milk Marketed in Zaria, Nigeria. Master’s Thesis.

[29] Igbinosa E.O., Odjadjare E.E., Igbinosa I.H., Orhue P.O., Omoigberale M.N., Amhanre N.I. (2012). Antibiotic synergy interaction against multidrug-resistant *Pseudomonas aeruginosa* isolated from an abattoir effluent environment. Sci. World J..

[30] Akobi B., Aboderin O., Sasaki T., Shittu A. (2012). Characterization of *Staphylococcus aureus* isolates from faecal samples of the straw-coloured fruit bat (*Eidon helvum*) in Obafemi Awolowo University (OAU), Nigeria. BMC Microbiol..

[31] Ojo O.E., Awosile B., Agbaje M., Sonibare A.O., Oyekunle M.A., Kasali O.B. (2012). Quinolone resistance in bacterial isolates from chicken carcasses in Abeokuta, Nigeria: A retrospective study from 2005–2011. Niger. Vet. J..

[32] Fashae K., Hendriksen R.S. (2013). Diversity and antimicrobial susceptibility of Salmonella enterica serovars isolated from pig farms in Ibadan, Nigeria. Folia Microbiol. (Praha).

[33] Mailafia S., Michael O., Kwaja E. (2013). Evaluation of microbial contaminants and antibiogram of Nigerian paper currency notes (Naira) circulation in Gwagwalada, Abuja, Nigeria. Niger. Vet. J..

[34] Oluduro A.O. (2012). Antibiotic-resistant commensal *Escherichia coli* in faecal droplets from bats and poultry in Nigeria. Vet. Ital..

[35] Kawo A.H., Musa A.M. (2013). Enumeration, isolation and antibiotic susceptibility profile of bacteria associated with mobile cellphones in a university environment. Niger. J. Basic Appl. Sci..

[36] Ogunleye A.O., Ajuwape A.T.P., Adetosoye A.I., Carlson S.A. (2013). Characterization of *Salmonella enterica* Ituri isolated from diseased poultry in Nigeria. Afr. J. Biotechnol..

[37] Oviasogie F.E., Agbonlahor D.E. (2013). The burden, antibiogram and pathogenicity of bacteria found in municipal waste dumpsites and on waste site workers in Benin City. J. Biomed. Sci..

[38] Raufu I., Bortolaia V., Svendsen C.A., Ameh J.A., Ambali A.G., Aarestrup F.M., Hendriksen R.S. (2013). The first attempt of an active integrated laboratory-based Salmonella surveillance programme in the North-eastern region of Nigeria. J. Appl. Microbiol..

[39] Suleiman A., Zaria L.T., Grema H.A., Ahmadu P. (2013). Antimicrobial resistant coagulase positive *Staphylococcus aureus* from chickens in Maiduguri, Nigeria. Sokoto J. Vet. Sci..

[40] Adelowo O.O., Fagade O.E., Agersø Y. (2014). Antibiotic resistance and resistance genes in *Escherichia coli* from poultry farms, southwest Nigeria. J. Infect. Dev. Ctries..

[41] Adefarakan T.A., Oluduro A.O., David O.M., Ajayi A.O., Ariyo A.B., Fashina C.D. (2014). Prevalence of antibiotic resistance and molecular characterization of *Escherichia coli* from faeces of apparently healthy rams and goats in Ile-Ife, Southwest, Nigeria. Ife J. Sci..

[42] Adeyanju G.T., Ishola O. (2014). *Salmonella* and *Escherichia coli* contamination of poultry meat from a processing plant and retail markets in Ibadan, Oyo State, Nigeria. Springerplus.

[43] Raufu I.A., Fashae K., Ameh J.A., Ambali A., Ogunsola F.T., Coker A.O., Hendriksen R.S. (2014). Persistence of fluoroquinolone-resistant Salmonella enterica serovar Kentucky from poultry and poultry sources in Nigeria. J. Infect. Dev. Ctries..

[44] Raufu I.A., Odetokun I.A., Oladunni F.S., Adam M., Kolapo U.T., Akorede G.J., Ghali I.M., Ameh J.A., Ambali A. (2015). Serotypes, antimicrobial profiles, and public health significance of Salmonella from camels slaughtered in Maiduguri central abattoir, Nigeria. Vet. World.

[45] Adenipekun E.O., Jackson C.R., Oluwadun A., Iwalokun B.A., Frye J.G., Barrett J.B., Hiott L.M., Woodley T.A. (2015). Prevalence and antimicrobial resistance in *Escherichia coli* from food animals in Lagos, Nigeria. Microb. Drug Resist..

[46] Olowe O.A., Adewumi O., Odewale G., Ojurongbe O., Adefioye O.J. (2015). Phenotypic and molecular characterisation of extended-spectrum Beta-lactamase producing *Escherichia coli* obtained from animal fecals Samples in Ado Ekiti, Nigeria. J. Environ. Public Health.

[47] Olonitola O.S., Fahrenfeld N., Pruden A. (2015). Antibiotic resistance profiles among mesophilic aerobic bacteria in Nigerian chicken litter and associated antibiotic resistance genes1. Poult. Sci..

[48] Ugwu C.C., Gomez-Sanz E., Agbo I.C., Torres C., Chah K.F. (2015). Characterization of mannitol-fermenting methicillin-resistant staphylococci isolated from pigs in Nigeria. Braz. J. Microbiol..

[49] Ayeni F.A., Odumosu B.T., Oluseyi A.E., Ruppitsch W. (2016). Identification and prevalence of tetracycline resistance in enterococci isolated from poultry in Ilishan, Ogun State, Nigeria. J. Pharm. Bioallied Sci..

[50] Eghomwanre A.F., Obayagbona N.O., Osarenotor O., Enagbonma B.J. (2016). Evaluation of antibiotic resistance patterns and heavy metals tolerance of some bacteria isolated from contaminated soils and sediments from Warri, Delta State, Nigeria. J. Appl. Sci. Environ. Manag..

[51] Igbinosa E.O. (2016). Detection and antimicrobial resistance of Vibrio isolates in aquaculture environments: Implications for public health. Microb. Drug Resist..

[52] Ishola O.O., Mosugu J.I., Adesokan H.K. (2016). Prevalence and antibiotic susceptibility profiles of *Listeria monocytogenes* contamination of chicken flocks and meat in Oyo State, south-western Nigeria: Public health implications. J. Prev. Med. Hyg..

[53] Ngbede E.O., Raji M.A., Kwanashie C.N., Kwaga J.K. (2017). Antimicrobial resistance and virulence profile of enterococci isolated from poultry and cattle sources in Nigeria. Trop. Anim. Health Prod..

[54] Odumosu B.T., Ajetumobi O., Adegbola H.D., Odutayo I. (2016). Antibiotic susceptibility pattern and analysis of plasmid profiles of *Pseudomonas aeruginosa* from human, animal and plant sources. SpringerPlus.

[55] Umaru G.A., Kwaga J.K.P., Bello M., Raji M.A., Maitala Y.S. (2016). Antibiotic resistance of *Staphylococcus aureus* isolated from fresh cow milk in settled Fulani herds in Kaduna State, Nigeria. Bull. Anim. Health Prod. Afr..

[56] Dipeolu M.A., Alonge D.O. (2001). Residues of tetracycline antibiotic in cattle meat marketed in Ogun and Lagos States of Nigeria. ASSET.

[57] Kabir J., Umoh J.U., Umoh V.J. (2002). Characterisation and screening for antimicrobial substances of slaughtered cattle in Zaria, Nigeria. Meat Sci..

[58] Dipeolu M.A. (2002). Residues of tetracycline antibiotic in marketed goats and pigs in Lagos and Ogun States Nigeria. Niger. J. Anim. Sci..

[59] Dipeolu M.A., Dada K.O. (2005). Residues of tetracycline in imported frozen chickens in South West Nigeria. Trop. Vet..

[60] Olatoye I.O., Ehinmowo A.A. (2010). Oxytetracycline residues in edible tissues of cattle slaughtered in Akure, Nigeria. Niger. Vet. J..

[61] Ezenduka E.V., Oboegbulem S.I., Nwanta J.A., Onunkwo J.I. (2011). Prevalence of antimicrobial residues in raw table eggs from farms and retail outlets in Enugu State, Nigeria. Trop. Anim. Health Prod..

[62] Adetunji V.O., Olaoye O.O. (2012). Detection of β-Lactam antibiotics (Penicillin and Amoxicillin) residues in Goat milk. Nat. Sci..

[63] Olatoye O., Kayode S.T. (2012). Oxytetracycline residues in retail chicken eggs in Ibadan, Nigeria. Food Addit. Contam. Part B Surveill..

[64] Ezenduka E.V., Ugwumba C. (2012). Antimicrobial residues screening in pigs and goats slaughtered in Nsukka Municipal abattoir, Southeast Nigeria. Afr. J. Biotechnol..

[65] Omeiza K.G., Otopa E.A., Okwoche J.O. (2012). Assessment of drug residues in beef in Abuja, the Federal Capital Territory, Nigeria. Vet. Ital..

[66] Olatoye I.O., Oyelakin E.F., Adeyemi I.G., Call D.R. (2012). Chloramphenicol use and prevalence of its residues in broiler chickens and eggs in Ibadan, Nigeria. Niger. Vet. J..

[67] Olusola A.V., Folashade P.A., Ayoade O.I. (2012). Heavy metal (lead, Cadmium) and antibiotic (Tetracycline and Chloramphenicol) residues in fresh and frozen fish types (*Clarias gariepinus*, *Oreochromis niloticus*) in Ibadan, Oyo State, Nigeria. Pak. J. Biol. Sci..

[68] Olusola A.V., Diana B.E., Ayoade O.I. (2012). Assessment of tetracycline, lead and cadmium residues in frozen chicken vended in Lagos and Ibadan, Nigeria. Pak. J. Biol. Sci..

[69] Adesokan H.K., Agada C.A., Adetunji V.O., Akanbi I.M. (2013). Oxytetracycline and penicillin-G residues in cattle slaughtered in south-western Nigeria: Implications for livestock disease management and public health. J. S. Afr. Vet. Assoc..

[70] Mbodi F.E., Nguku P., Okolocha E., Kabir J. (2014). Determination of chloramphenicol residues in commercial chicken eggs in the Federal Capital Territory, Abuja, Nigeria. Food Addit. Contam. Part A Chem. Anal. Control Expo. Risk Assess..

[71] Olatoye I.O., Daniel O.F., Ishola S.A. (2015). Screening of antibiotics and chemical analysis of penicillin residue in fresh milk and traditional dairy products in Oyo state, Nigeria. Vet. World.

[72] Adesida S.A., Coker A.O., Smith S.I. (2003). Resistotyping of *Campylobacter jejuni*. Niger. Postgrad. Med. J..

[73] Moher D., Liberati A., Tetzlaff J., Altman D.G., The PRISMA Group (2009). Preferred reporting items for systematic reviews and meta-analyses: The PRISMA statement. PLoS Med..

[74] Coates A.R., Halls G., Hu Y. (2011). Novel classes of antibiotics or more of the same?. Br. J. Pharmacol..

[75] World Organization for Animal Health (2015). List of Antimicrobial Agents of Veterinary Importance. http://www.oie.int/fileadmin/Home/eng/Our_scientific_expertise/docs/pdf/Eng_OIE_List_antimicrobials_May2015.pdf.

[76] World Health Organization Critically Important Antimicrobials for Human Medicine. 5th Revision 2016. Ranking of Medically Important Antimicrobials for Risk Management of Antimicrobial Resistance Due to Non-Human Use. http://apps.who.int/iris/bitstream/10665/255027/1/9789241512220-eng.pdf.

[77] Israel E.U., Emmanuel E.G., Sylvester E.G., Chukwuma E. (2015). Self-medication with antibiotics amongst Civil Servants in Uyo, Southern Nigeria. J. Adv. Med. Pharm. Sci..

[78] Federal Ministries of Agriculture and Rural Development, Environment, and Health (2017). Antimicrobial Use and Resistance in Nigeria: Situation Analysis and Recommendations. http://www.ncdc.gov.ng/themes/common/docs/protocols/56_1510840387.pdf.

[79] Adesokan H., Akanbi I., Akanbi I., Obaweda R. (2015). Pattern of antimicrobial usage in livestock animals in south-western Nigeria: The need for alternative plans. Onderstepoort J. Vet. Res..

[80] World Organization for Animal Health (2017). OIE Terrestrial Animal Health Code. http://www.rr-africa.oie.int/docspdf/en/Codes/en_csat-vol1.pdf.

