# Supplementary figures and images for: Antimicrobial Resistance in Food Animals and the Environment in Nigeria: A Review
Source: Int J Environ Res Public Health. 2018 Jun 17;15(6):1284. doi: 10.3390/ijerph15061284 (PMC6025306; doi:10.3390/ijerph15061284)

Figure S1

Figure S1: Flow chart of the methodological strategy (Modified PRISMA 2009 Flow Diagram)

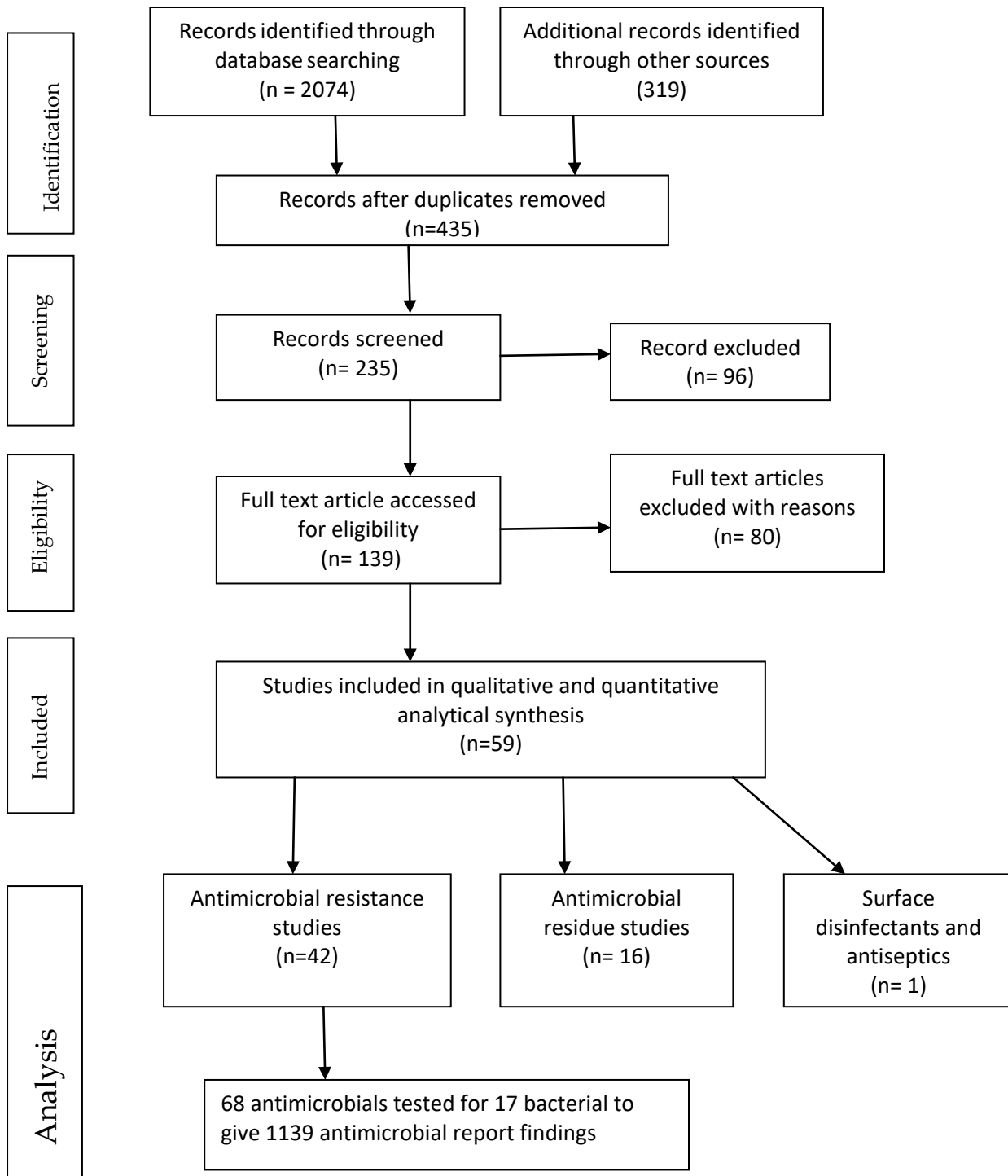

Supplement: Supplementary file 1 [file ijerph-15-01284-s001.zip › Suppl/Figure S1.pdf]

Figure S10

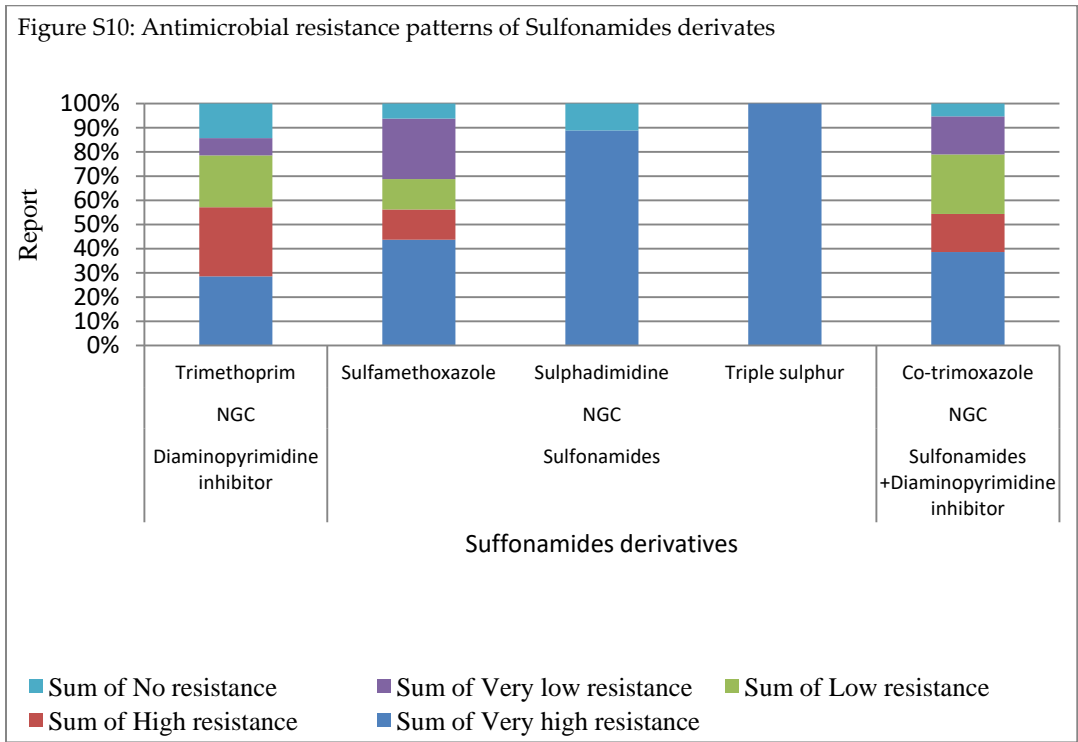

NGC= Non generational classification

Supplement: Supplementary file 1 [file ijerph-15-01284-s001.zip › Suppl/Figure S10.pdf]

Figure S11

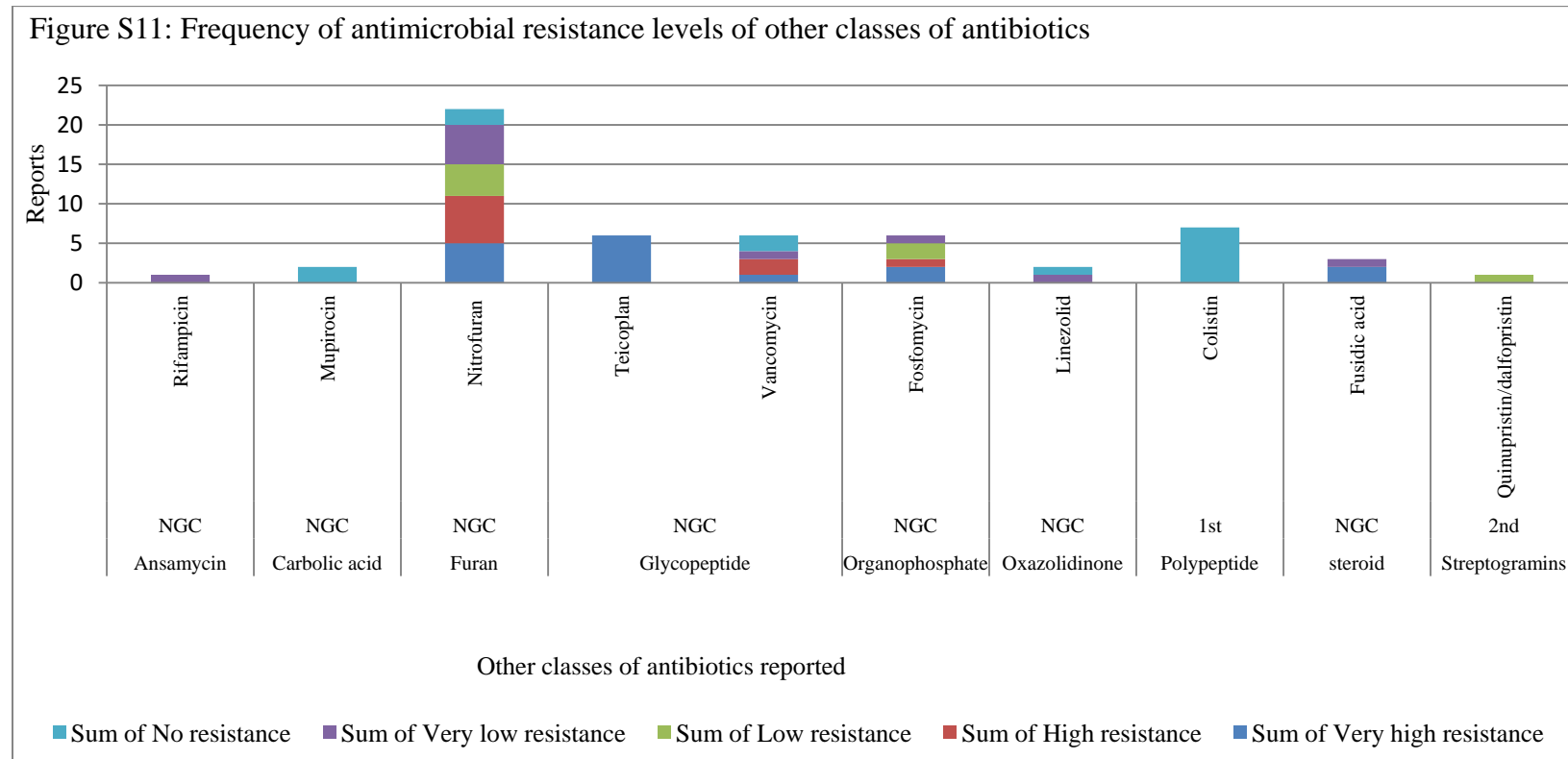

Supplement: Supplementary file 1 [file ijerph-15-01284-s001.zip › Suppl/Figure S11.pdf]

Figure S12

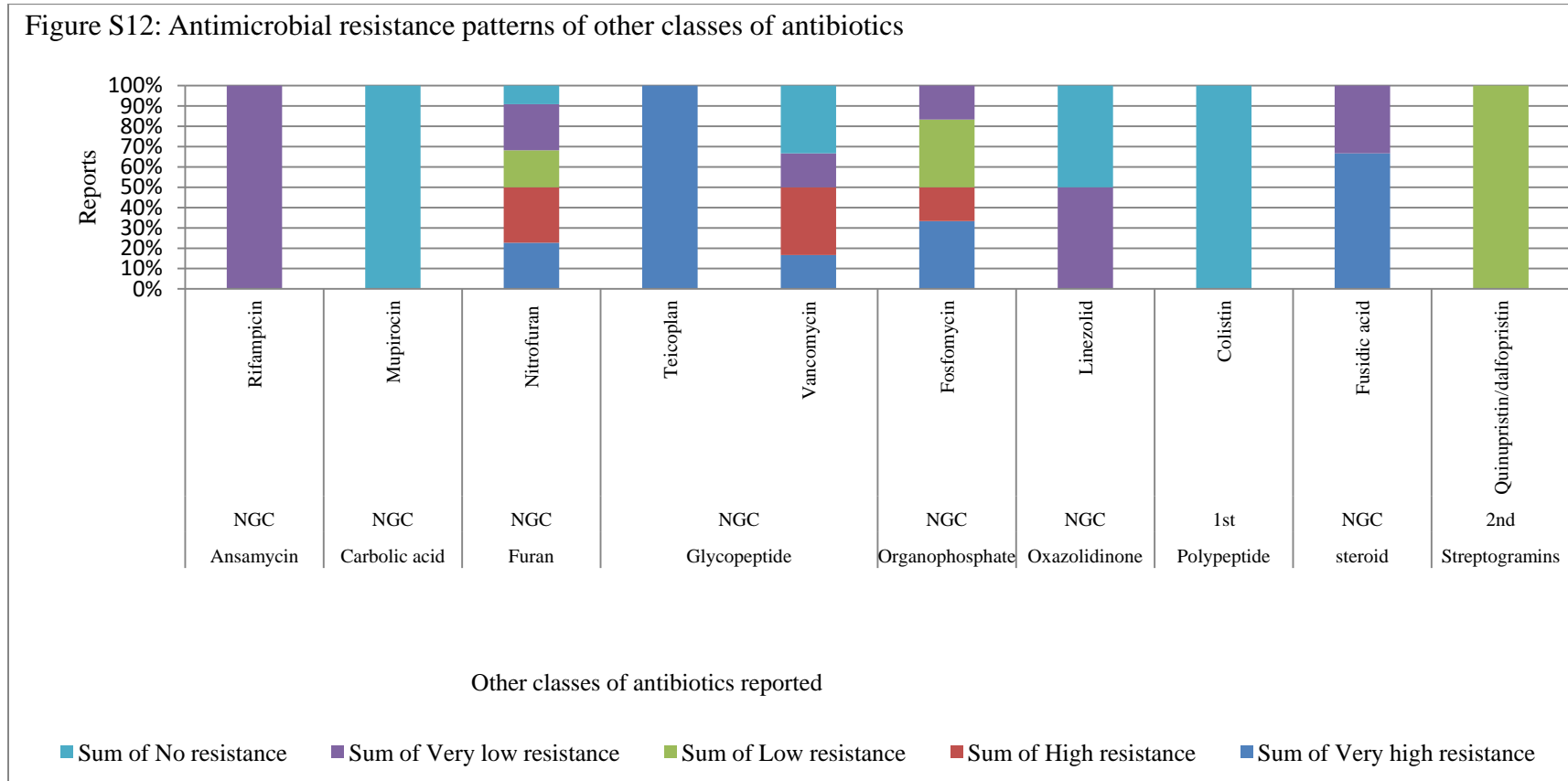

Supplement: Supplementary file 1 [file ijerph-15-01284-s001.zip › Suppl/Figure S12.pdf]

Figure S13

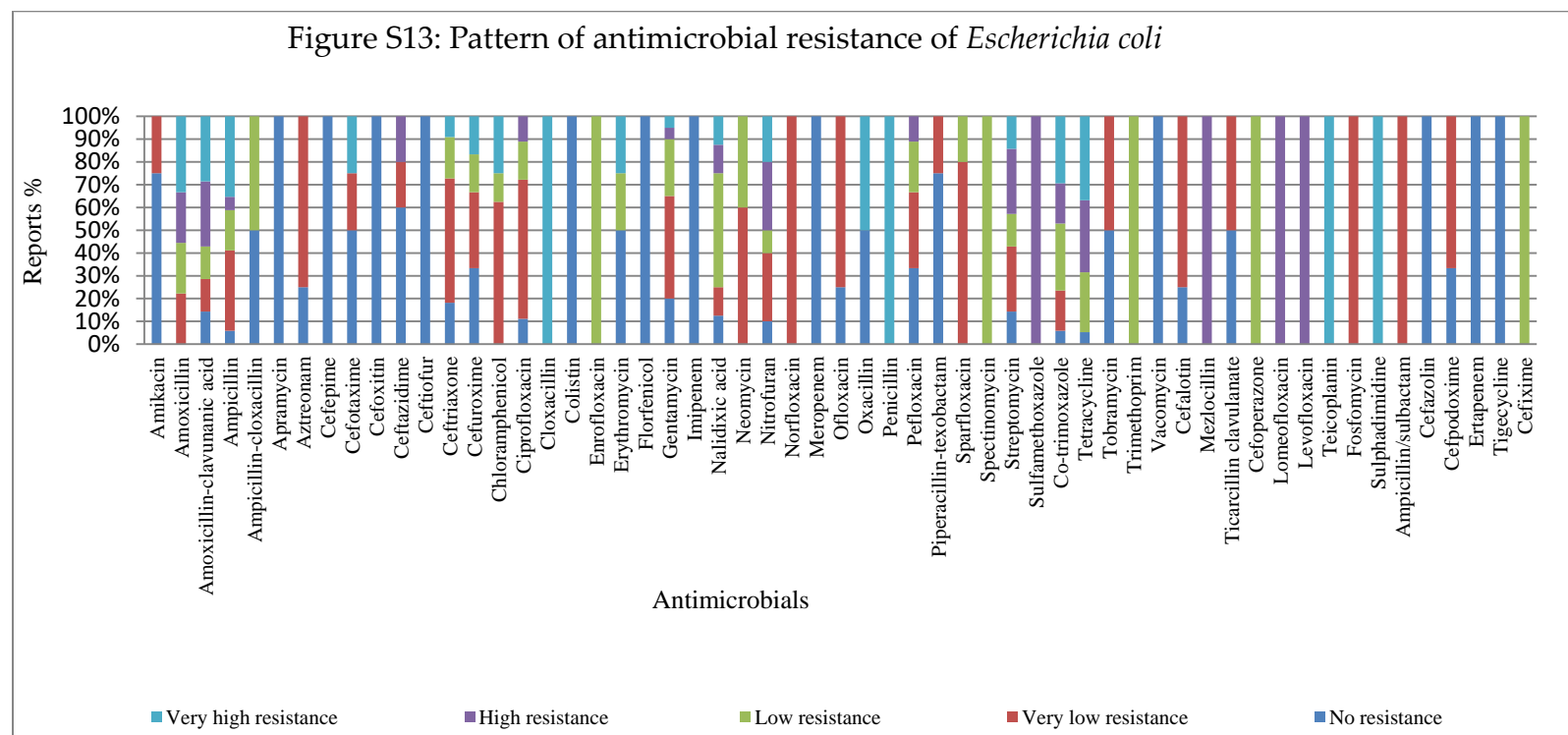

Supplement: Supplementary file 1 [file ijerph-15-01284-s001.zip › Suppl/Figure S13.pdf]

Figure S14

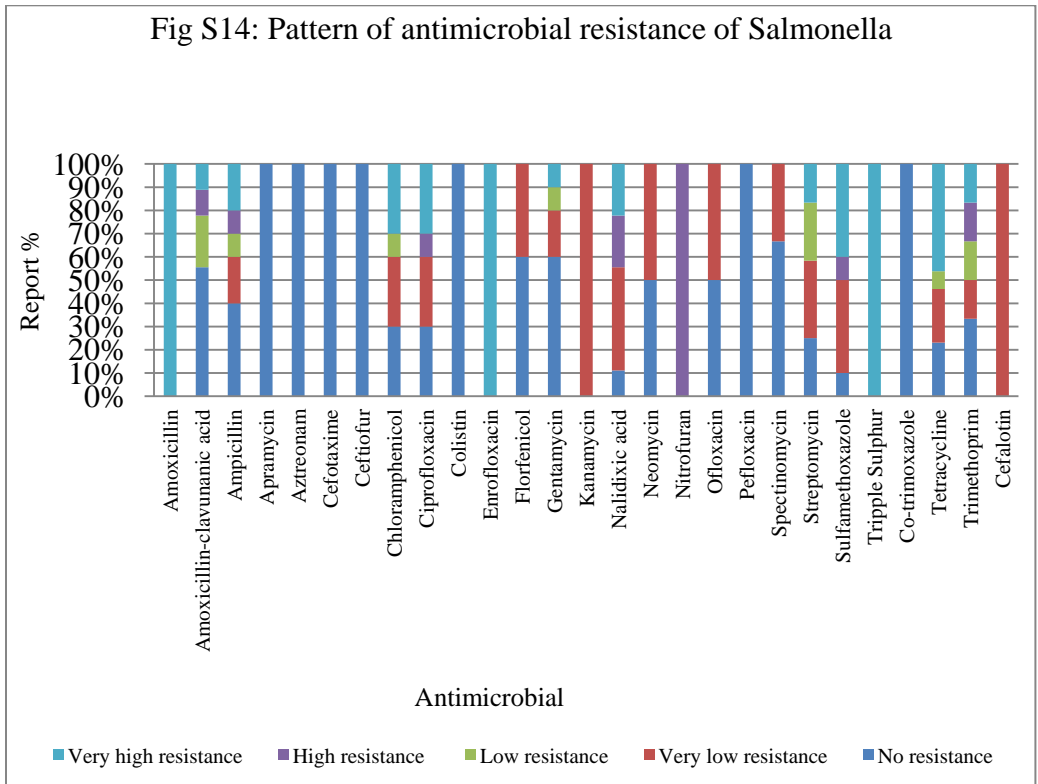

Supplement: Supplementary file 1 [file ijerph-15-01284-s001.zip › Suppl/Figure S14.pdf]

Figure S15

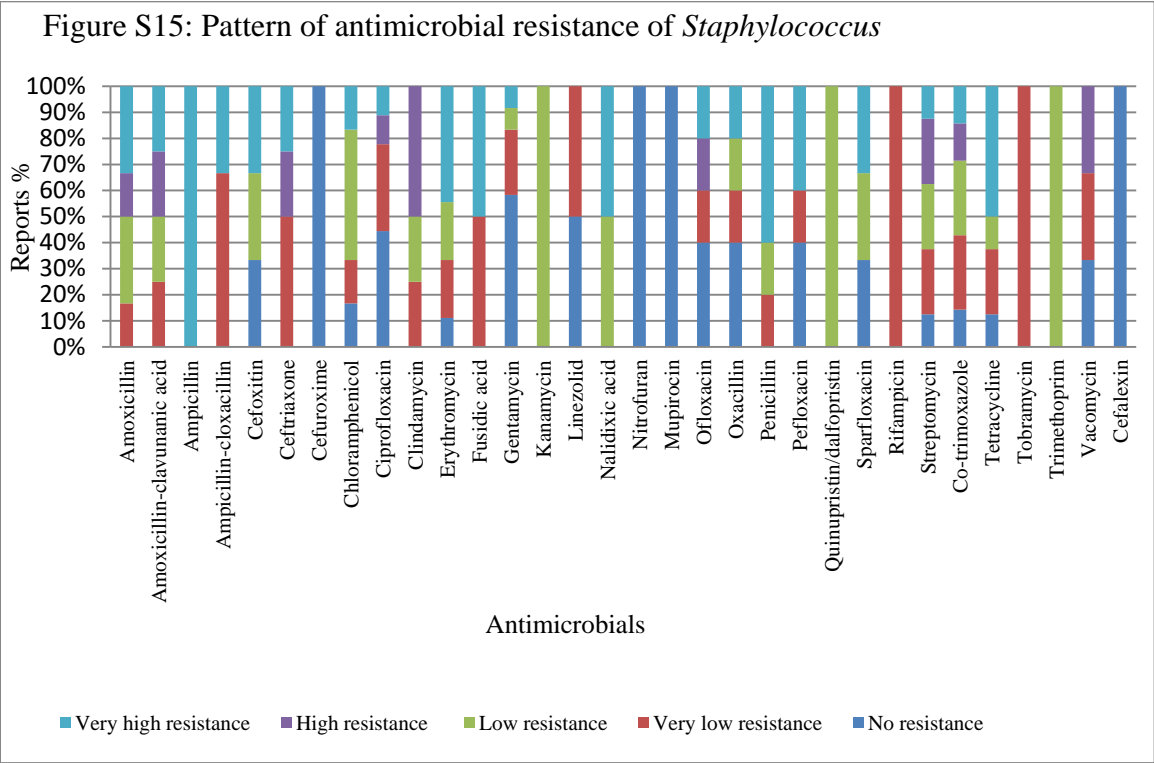

Supplement: Supplementary file 1 [file ijerph-15-01284-s001.zip › Suppl/Figure S15.pdf]

Figure S16

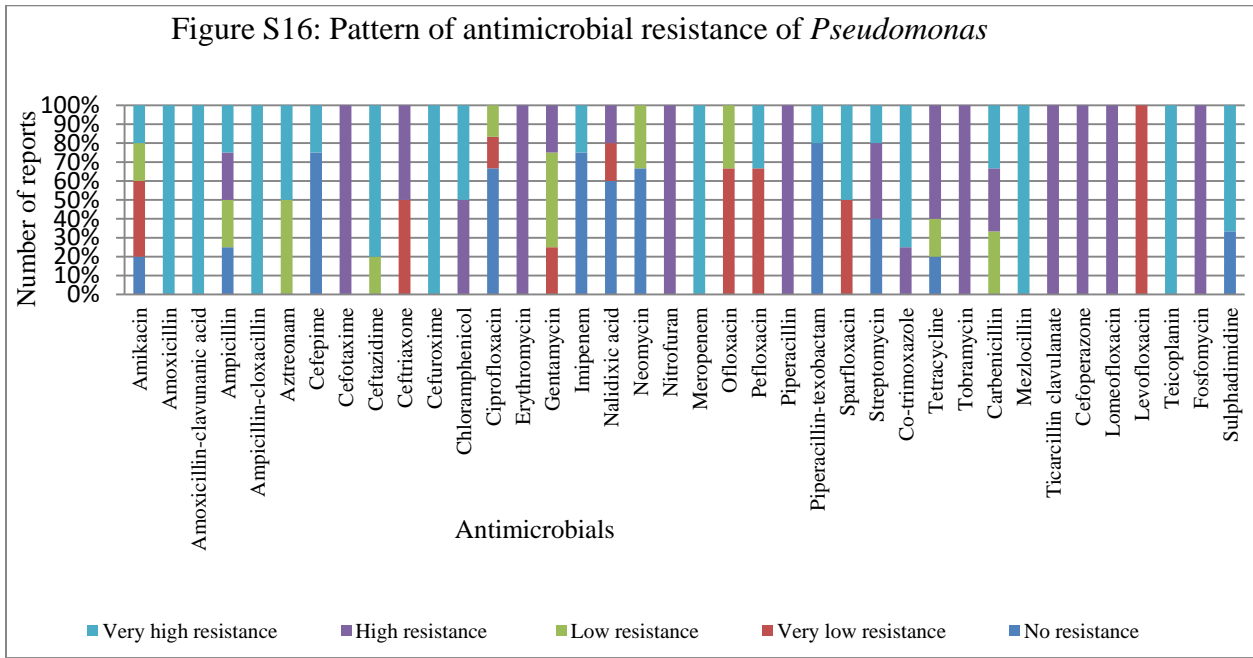

Supplement: Supplementary file 1 [file ijerph-15-01284-s001.zip › Suppl/Figure S16.pdf]

Figure S17

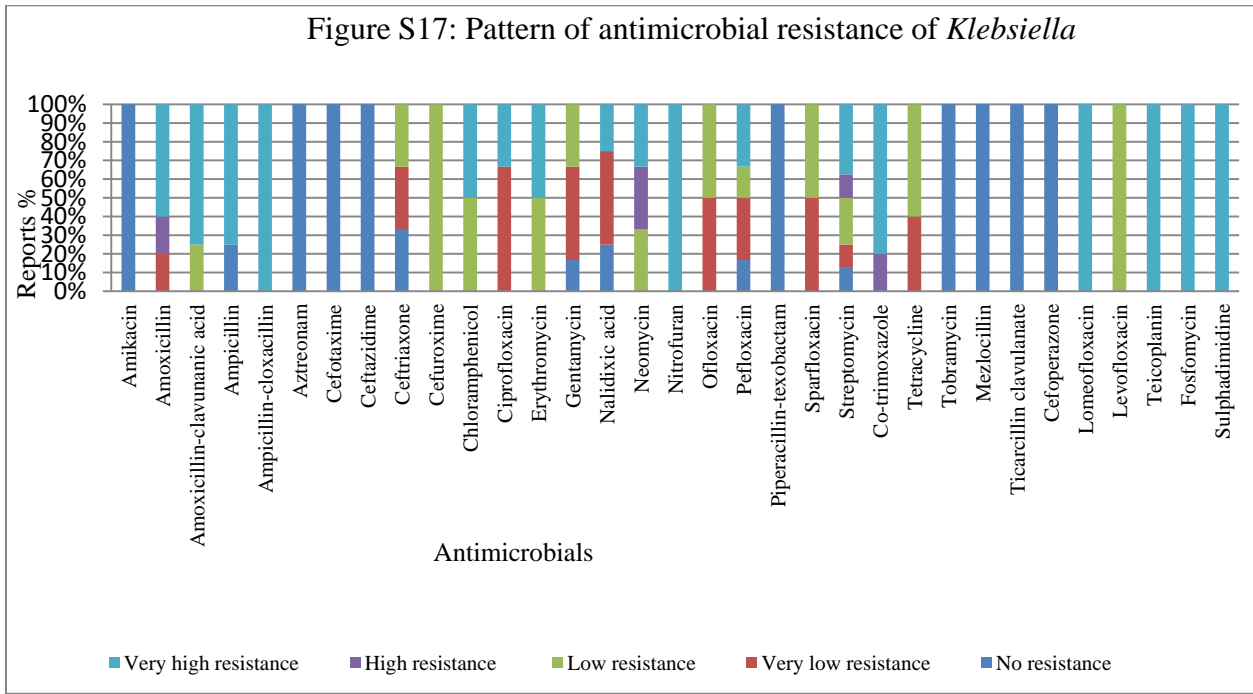

Supplement: Supplementary file 1 [file ijerph-15-01284-s001.zip › Suppl/Figure S17.pdf]

Figure S18

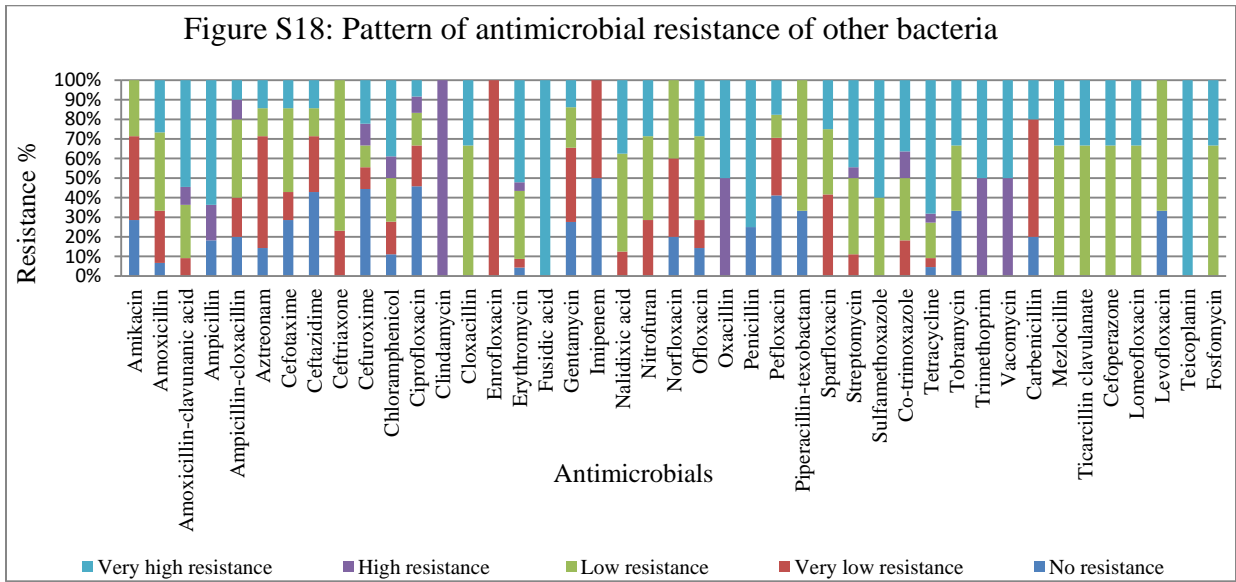

Supplement: Supplementary file 1 [file ijerph-15-01284-s001.zip › Suppl/Figure S18.pdf]

Figure S2

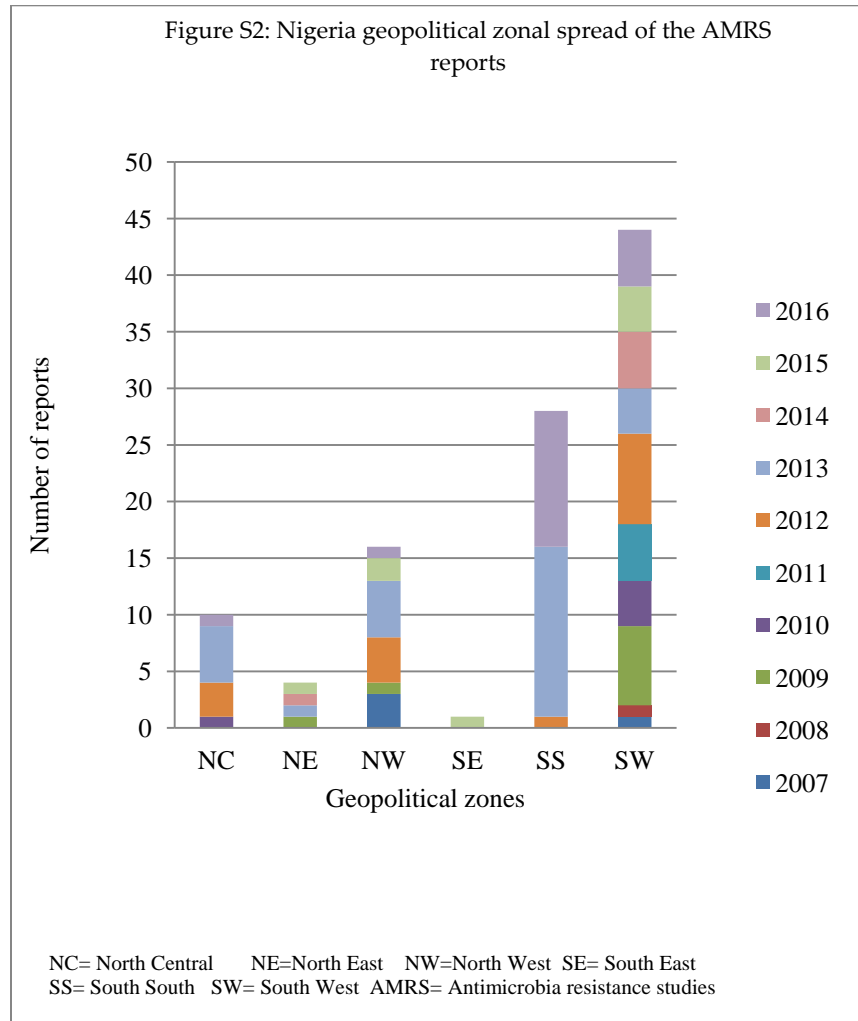

Supplement: Supplementary file 1 [file ijerph-15-01284-s001.zip › Suppl/Figure S2.pdf]

Figure S3

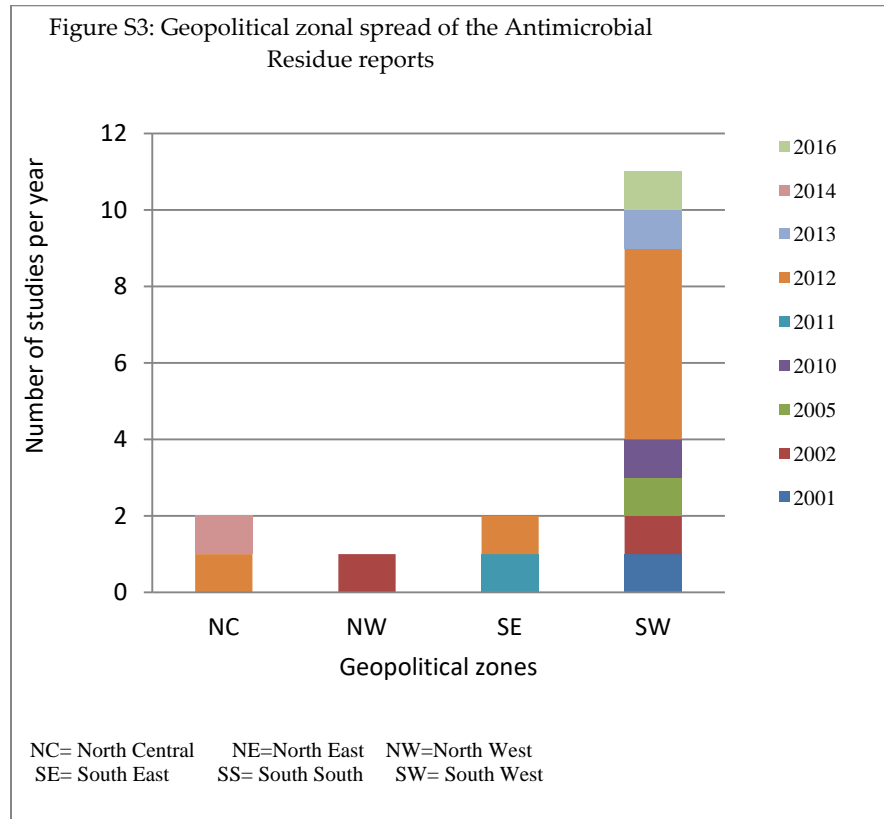

Supplement: Supplementary file 1 [file ijerph-15-01284-s001.zip › Suppl/Figure S3.pdf]

Figure S4a

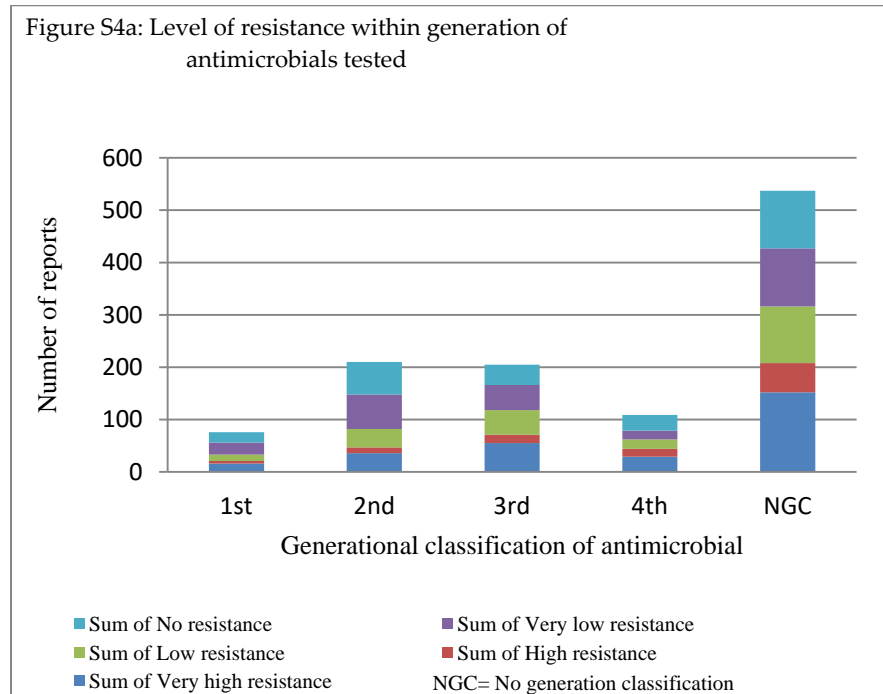

Supplement: Supplementary file 1 [file ijerph-15-01284-s001.zip › Suppl/Figure S4a.pdf]

Figure S4b

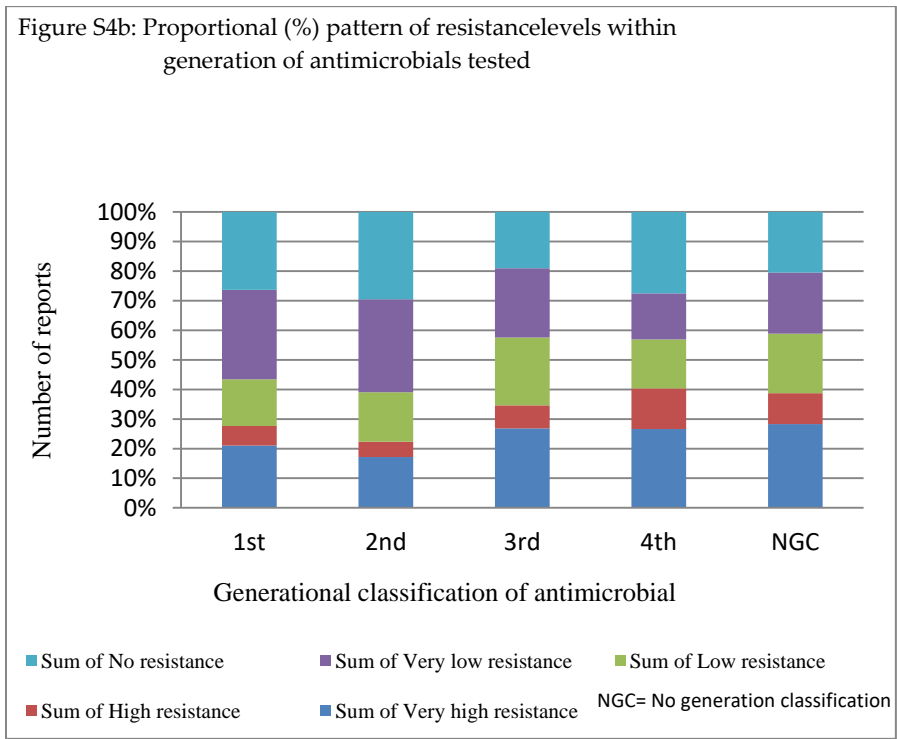

Supplement: Supplementary file 1 [file ijerph-15-01284-s001.zip › Suppl/Figure S4b.pdf]

Figure S5a

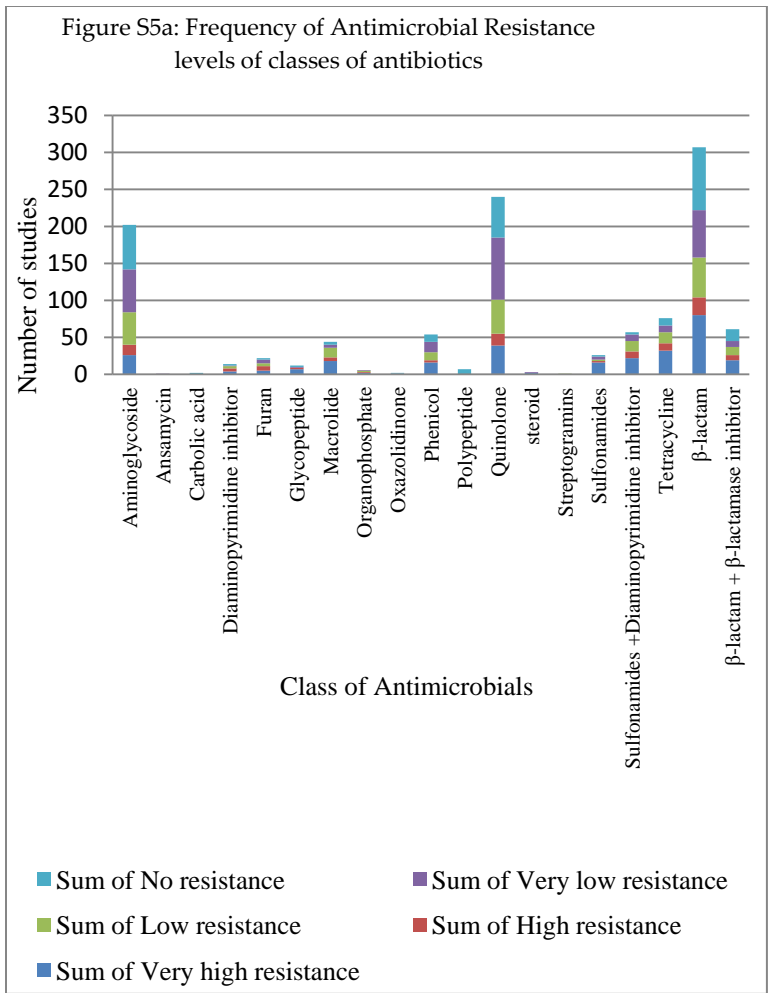

Supplement: Supplementary file 1 [file ijerph-15-01284-s001.zip › Suppl/Figure S5a.pdf]

Figure S5b

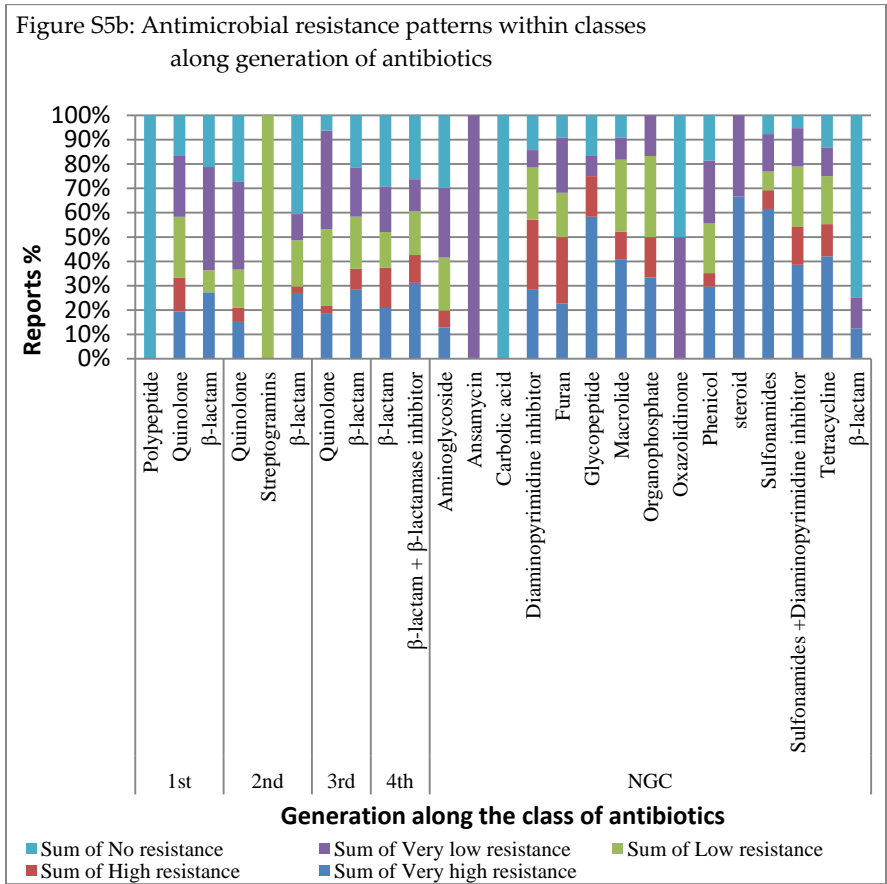

Supplement: Supplementary file 1 [file ijerph-15-01284-s001.zip › Suppl/Figure S5b.pdf]

Figure S6

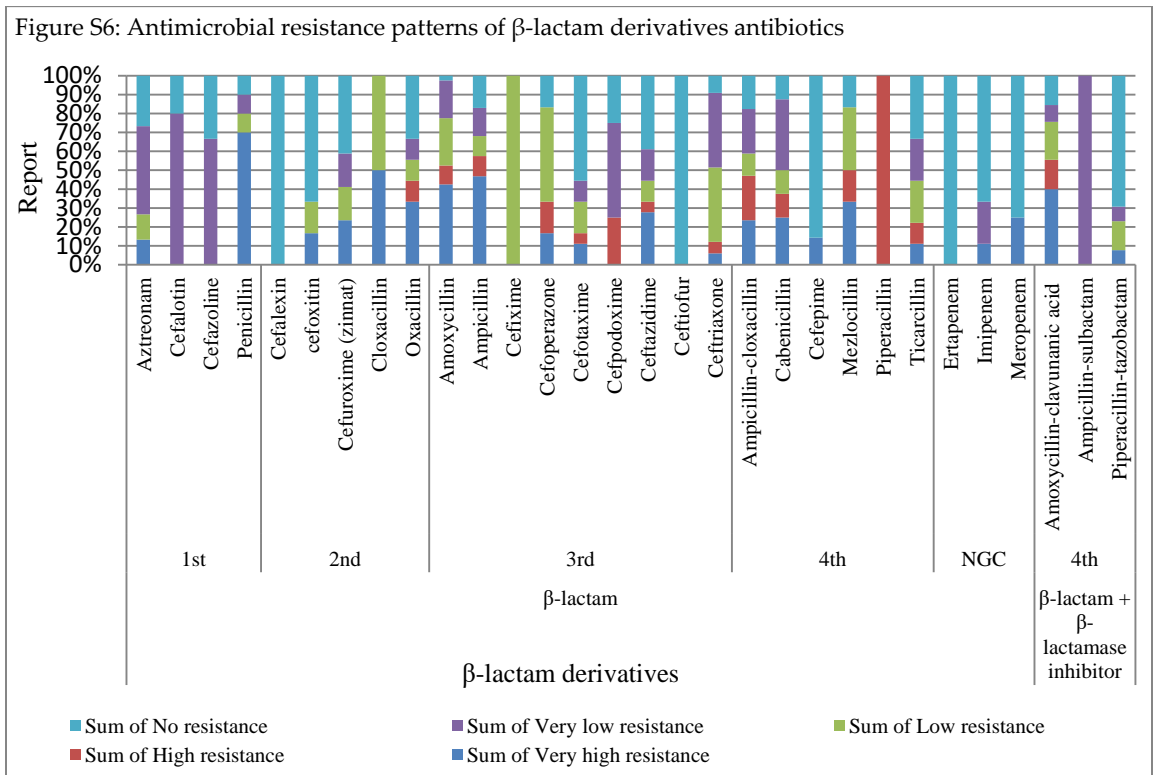

Supplement: Supplementary file 1 [file ijerph-15-01284-s001.zip › Suppl/Figure S6.pdf]

Figure S7

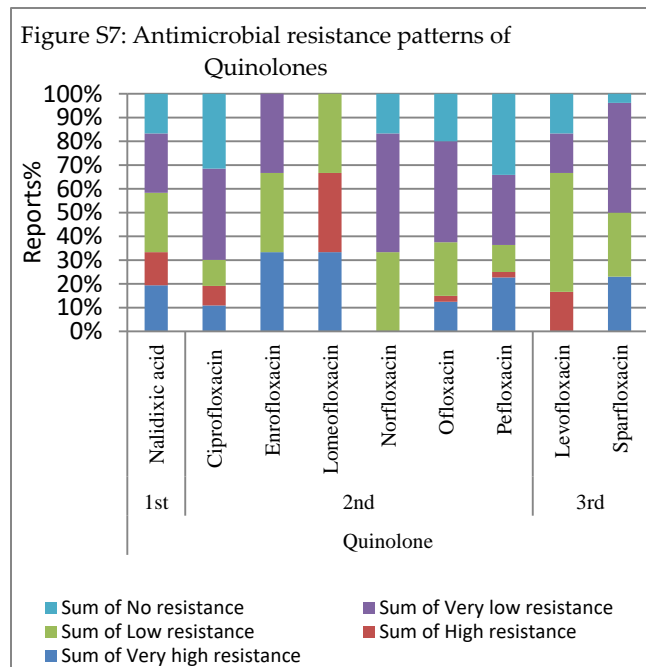

Supplement: Supplementary file 1 [file ijerph-15-01284-s001.zip › Suppl/Figure S7.pdf]

Figure S8

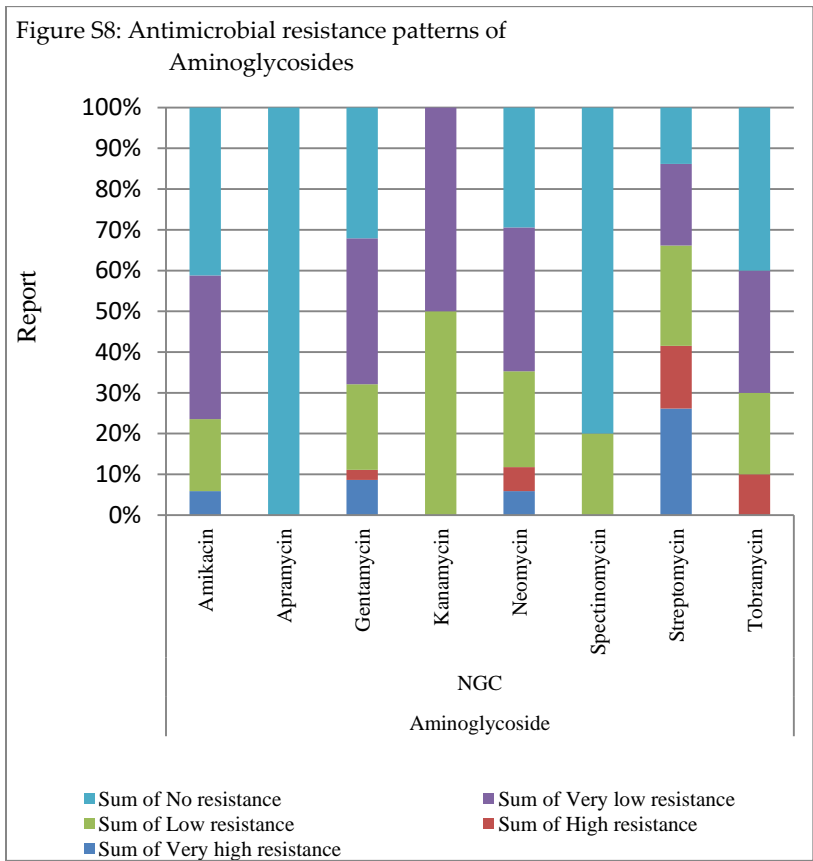

Supplement: Supplementary file 1 [file ijerph-15-01284-s001.zip › Suppl/Figure S8.pdf]

Figure S9

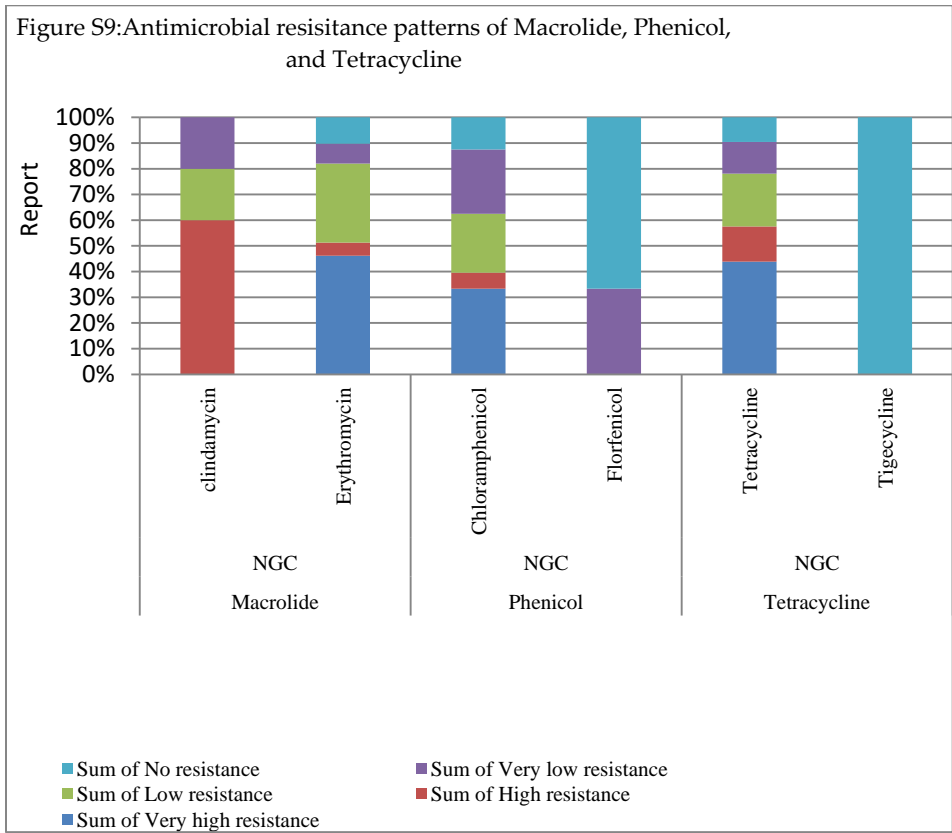

Supplement: Supplementary file 1 [file ijerph-15-01284-s001.zip › Suppl/Figure S9.pdf]
